# Oxygen Functionalization-Induced Charging Effect on Boron Active Sites for High-Yield Electrocatalytic NH_3_ Production

**DOI:** 10.1007/s40820-022-00966-7

**Published:** 2022-11-05

**Authors:** Ashmita Biswas, Samadhan Kapse, Ranjit Thapa, Ramendra Sundar Dey

**Affiliations:** 1 Institute of Nano Science and Technology (INST), Sector-81, Mohali, Punjab 140306 India; 2 Department of Physics, SRM University–AP, Amaravati, Andhra Pradesh 522240 India

**Keywords:** C-doped boron nitride, O-functionalization, Density-functional theory (DFT), Charging effect, Nitrogen reduction reaction, Ambient ammonia synthesis

## Abstract

Ammonia has been recognized as the future renewable energy fuel because of its wide-ranging applications in H_2_ storage and transportation sector. In order to avoid the environmentally hazardous Haber–Bosch process, recently, the third-generation ambient ammonia synthesis has drawn phenomenal attention and thus tremendous efforts are devoted to developing efficient electrocatalysts that would circumvent the bottlenecks of the electrochemical nitrogen reduction reaction (NRR) like competitive hydrogen evolution reaction, poor selectivity of N_2_ on catalyst surface. Herein, we report the synthesis of an oxygen-functionalized boron carbonitride matrix via a two-step pyrolysis technique. The conductive BNCO_(1000)_ architecture, the compatibility of B-2*p*_z_ orbital with the N-2*p*_z_ orbital and the charging effect over B due to the C and O edge-atoms in a pentagon altogether facilitate N_2_ adsorption on the B edge-active sites. The optimum electrolyte acidity with 0.1 M HCl and the lowered anion crowding effect aid the protonation steps of NRR via an associative alternating pathway, which gives a sufficiently high yield of ammonia (211.5 μg h^−1^ mg_cat_^−1^) on the optimized BNCO_(1000)_ catalyst with a Faradaic efficiency of 34.7% at − 0.1 V vs RHE. This work thus offers a cost-effective electrode material and provides a contemporary idea about reinforcing the charging effect over the secured active sites for NRR by selectively choosing the electrolyte anions and functionalizing the active edges of the BNCO_(1000)_ catalyst.

**Supplementary Information:**

The online version contains supplementary material available at 10.1007/s40820-022-00966-7.

## Highlights

- O-functionalization at the edges of boron carbonitride induces charge polarization effect on B.
- 0.1 M HCl serves to preserve the catalyst active site from poisoning effect by electrolyte anions.
- Experimental and theoretical findings go hand-in-hand towards high yield of ammonia.

**Supplementary Information:**

The online version contains supplementary material available at 10.1007/s40820-022-00966-7.

## Graphical abstract

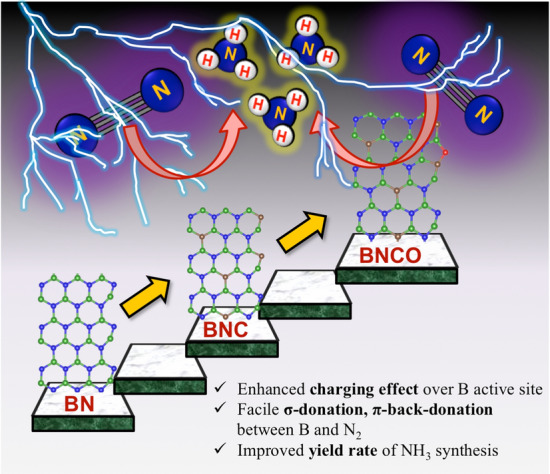

## Introduction

Nitrogen reduction reaction (NRR) performed electrochemically is regarded as a green and legitimate approach of ammonia synthesis and it has been intrinsically into limelight over the world-wide research community, not only because of the immense use of ammonia in the agriculture and transportation sector, but also due to urge to resolve the fallacies associated with the process [1–3]. Primarily, the eternal problem persisting with NRR is the predominance of the combative hydrogen evolution reaction (HER) at the same potential range, which overpowers NRR over most of the catalyst surfaces, resulting in poor yield and Faradaic efficiency (FE) of ammonia production. Researchers thus majorly focus on varied catalyst development, which includes several strategies: (a) Selectivity of elements that would prefer binding with N_2_ over protons [4–6], (b) Blocking the HER active sites [7], (c) Phase-selective material designing, inhibiting HER at the active surface [8, 9], (d) interface-engineering that would deviate the HER pathway inducing better Faradaic efficiency for NRR [10]. Although either 1^st^ row transition metals [11] or semimetals [12] are regarded as more suitable candidates for NRR, a metal-free approach is rather preferred for the cost-effectiveness and simplicity of the process. Boron (B)-based electrocatalyst in this respect can act as a strong contender [12, 13]

The endless advantages of boron nitride (BN), where B serves as a Lewis acidic site and its orbital compatibility with N_2_, which is on the other hand a weak Lewis base appears to serve the purpose of N_2_ adsorption on the B units successfully. However, in order to obtain a better yield of ammonia, it is crucial to drive the forces that tend to bind N_2_ with B active sites that is the σ-bonding and the π-back bonding interactions that weaken the N≡N bond on the catalyst surface [14]. One probable way out to this could be doping some foreign elements in the BN architecture that would either serve as a better active site for NRR [15] or enhance the local charging effect over B to make way for the delocalization of the charges to the adsorbed N_2_ for the facile increment of N–N bond length and easy first protonation [16]. C-doping in BN framework is looked upon for this purpose, where some reports suggest that BN acts as a trigger while the edge C provided the active sites for NRR [17]. Contrary to this, a few reports considered B as the active centre in the BNC or B-doped C catalyst accomplishing facile N_2_ adsorption and reduction with a knee-high energy requirement [18, 19]. In particular, the edge B atoms having lone pair of electrons were found to be more effective to draw N_2_ for adsorption. However, there is a lagging in parity between theoretical and experimental findings to establish the actual active site and the importance of C in the BN framework, promoting NRR. It is more interesting to observe that in order to increase the efficiency of metal-free carbon-based catalyst, heteroatoms like O play crucial role to accumulate free charge cloud over the adjacent atoms and help in spin polarization, which in turn lower the potential for the rate determining steps of NRR [20, 21]. Thus, this concept could be rendered in BCN class of catalysts to provoke the NRR efficiency of the B active sites through charging effect, however, has not been explored.

Besides the active site, electrolytes play a parallel importance in NRR. Mostly in NRR, there is no selection rule for the choice of electrolytes. It has been seen in many cases that electrolyte ions (cations or anions) play a major role to provoke NRR [22, 23], either by suppressing HER [24] or by enhancing the local concentration of N_2_ in the vicinity of the electrode material [25]. However, the fact that electrolyte ions could also have some interaction with the active site is mostly unseen, as (a) This calls for a common practice to perform NRR in all the known electrolytes and (b) This requires rigorous theoretical findings. But along with the development of a suitable catalyst, the choice of electrolyte also needs thorough attention to ensure that the electrolyte ions do not offer poisoning effect on the active site.

In this work, we have insightfully focused on the above-mentioned issues of catalyst development and its preservation throughout the NRR process. Keeping in mind, the significant contribution of B towards NRR, our active catalyst BNCO_(1000)_ was developed. It was found that the presence of C dopants and O functionalities formed an electron-rich pentagon at the edges, deviating from the regular B–N–C hexagonal units owing to which the adjacent B site encountered a better charging effect and the material experienced improved conductivity with charge cloud density accumulated near the Fermi level. This helped to improve the charge transfer efficiency from B to the adsorbed N_2_ reinforcing the potential determining steps of NRR. More interestingly, a series of experiments with different set of acidic electrolytes like HCl, H_2_SO_4_ and H_3_PO_4_ (varying the anionic counterpart) revealed that HCl is a better competent for NRR on B active site yielding 211.5 μg h^−1^ mg_cat_^−1^ NH_3_ and with a FE of 34.7% at − 0.1 V vs RHE. H_2_SO_4_ and H_3_PO_4_ caused a crowding effect because of the bulkiness of anions hindering the passage of N_2_ and also bound with the B site through O end (SO_4_^2−^ and PO_4_^3−^ serving as better Lewis bases than N_2_), partly poisoning the active site. This study thus brings to the fore the importance of catalyst development as well as selectivity of electrolyte for an unperturbed and high yield of ammonia.

## Experimental and Calculations

### Material Synthesis

This work concerns the synthesis of the active material by a two-step pyrolysis method. Primarily, melamine and boric acid were taken as precursors of C, N, O and B in 1:2 weight ratio. After stirring the mixture in ethanol, it was dried overnight and the resultant powder was pyrolyzed at 550 °C followed by 1000 °C (3 °C min^−1^) for 2 h. Thereafter, the obtained black powder was subsequently washed thoroughly with distilled water and ethanol and dried in hot air oven at 70 °C to obtain oxygen edge-functionalized boron-carbonitride framework (BNCO_(1000)_). The other control samples were obtained at a different pyrolysis temperatures of 800, 900, and 1100 °C named as BNCO_(800)_, BNCO_(900)_, and BNCO_(1100)_, respectively, while maintaining the ratio of melamine and boric acid as 1:2. In order to establish the importance of B as an active site for NRR, only melamine was pyrolyzed at 1000 °C at 2 °C min^−1^ to obtain NC. In addition to this, to foreplay the effect of C doping, pristine BN was synthesized using boric acid and urea by pyrolyzing the precursors at 1000 °C for 5 h [26].

### Electrochemical Measurements

All the electrochemical characterizations involved in this work were carried out in an H-shaped electrolysis cell, where the cathodic and anodic compartments were separated by Nafion (115) membrane. This membrane allows permeability of protons from one chamber to the other and is non-selective to any other ions. The Nafion membrane was precleaned in 5 wt% H_2_O_2_ aqueous solution at 80 °C for 1 h followed by rinsing in ultrapure water at 80 °C for next 1 h [27]. All the measurements were ambiently taken at room temperature in an ideal three-electrode condition with Pt wire, Ag/AgCl (3 M KCl) and BNCO_(1000)_ modified GCE taken as the counter, reference and working electrodes, respectively. The cell chambers were immersed with 45 mL of the working electrolytes. The potentials for the reference electrode were related to the reversible hydrogen electrode (RHE) by Eq. 1 as follows:

1 $$E_{{{\text{RHE}}}} = E_{{{\text{Ag}}/{\text{AgCl}}}} + \, \left( {0.0{591 } \times {\text{ pH}}} \right) \, + \, 0.{21}0 \, (E^{0} {\text{at Ag}}/{\text{AgCl}},{\text{ 3 M KCl}})$$

Prior to each electrolysis, the electrolyte in the cathode compartment was continuously fed with pure Ar and N_2_ (99.99% purity) gases for 30 min each using properly positioned spargers so that the cathode could sufficiently get access to the gas bubbles. To ensure that pure N_2_ gas was fed into the electrolyte, it was passed subsequently through 0.05 M H_2_SO_4_ (acid trap) and 0.1 M KOH (base trap) for extracting any adventitious NH_3_ or NO_x_ present in the gas. All presented polarization curves were steady-state ones after 10 cycles and were measured at 10 mV s^−1^ scan rate and the current density values were normalized to geometric surface areas (0.07 cm^2^). Chronoamperometric (CA) tests were conducted in the N_2_-fed 0.1 M HCl solution for 2 h over the potential range from 0.0 to –0.4 V vs RHE. After each CA tests, measured amount of aliquot was taken out from the electrolyte and studied by indophenol blue and Watt and Chrisp methods for the qualitative detection of ammonia and hydrazine, respectively. For comparative study, ^1^H-NMR was also performed with the concentrated electrolyte solutions.

### Detection Methods of Ammonia and Hydrazine

Ammonia was detected following the conventional Indophenol Blue method with slight modifications. 5 mL of the aliquot solution was taken and added to 2 mL of 10 mg mL^−1^ Phenol solution in ethanol, followed by 0.2 mL of 0.5 wt% of C_5_FeN_6_Na_2_O (sodium nitroferricyanide) in water. The resulting solution was added with NaOH solution containing trisodium citrate as buffer, till the pH reached above 9. Finally, 0.1 mL of NaClO was added and the solution mixture was stored in dark for 2 h before UV–visible spectroscopic analysis at ~ 630 nm. The concentration of ammonia evolved in the reduction process was determined by a calibration plot (concentration vs absorbance) obtained from a set of solutions containing a known concentration of NH_4_Cl in 0.1 M HCl. To each of these solutions, the above-mentioned reagents were added and their absorbance was measured after a 2 h incubation time. The concentration of the produced NH_3_ was deduced following the equation *y* = 0.2523 *x* + 0.0508.

For hydrazine detection, the indicator solution contained 0.6 g of para-(dimethylamino) benzaldehyde in 30 mL absolute ethanol and 3 mL concentrated HCl (35%). 2 mL of this colour agent was mixed to same volume of the electrolyte solution and incubated in dark for 15 min before performing the UV–visible spectroscopic characterization. A set of solutions with known concentration of N_2_H_4_ in 0.1 M HCl was used as a calibration standard and their absorbance was measured at λ = 460 nm.

### Activity Descriptors

The NH_3_ yield rate (*R*_NH3_), normalized to mass, given by μg h^−1^ mg_cat_^−1^ can be calculated using Eq. 2, where *C* is the measured NH_3_ concentration (μg mL^−1^), *V* is the volume of the catholyte (mL), *t* is the electrolysis time (h), and mg_cat_ is the mass of the catalyst loaded on the electrode surface.

2 $$R = \frac{C \times V}{{t \times {\text{mg}}_{{{\text{cat}}}} }}$$

The Faradaic efficiency (FE) is calculated using Eq. 3, where 3 is the number of electrons necessary to produce one NH_3_ molecule, F is the Faraday constant (96,485 C mol^−1^), M is the relative molar mass of NH_3_ (M = 17 g mol^−1^), and the *Q* is the total charge passed through the electrodes (*C*).

3 $${\text{FE}} = \frac{3 \times F \times C \times V}{{M \times Q}}$$

The mass-normalized production rate of NH_3_ (mmol h^−1^ g_cat_^−1^) was calculated as Eq. 4 below:

4 $${\text{Production rate}}_{{{\text{mass}}}} = \frac{C \times V}{{M \times t \times g_{{cat^{ - 1} }} }}$$

### Quantification of NH_3_ Concentration from NMR

The catholyte solution was concentrated to 1 mL and 400 μL was taken out of it for NMR analysis. This was subsequently added with 50 μL of 0.01 M maleic acid solution followed by DMSO-d^6^ and subjected to ^1^H-NMR study. The obtained peaks were integrated and by using the following Eq. 5, the concentration of NH_3_ was quantified and matched with that obtained from UV–visible spectroscopic method.

5 $$\frac{{I_{{{\text{sample}}}} }}{{I_{{{\text{standard}}}} }} = \frac{{H_{{{\text{sample}}}} \times C_{{{\text{sample}}}} }}{{H_{{{\text{standard}}}} \times C_{{{\text{standard}}}} }}$$

where *I* stands for the integral values, H stands for the number of protons (4 in case of sample NH_4_^+^ and 2 in case of the vinylic protons of maleic acid) and *C* stands for the concentrations of the sample and standard (0.01 M for maleic acid).

### Electrochemical Active Surface Area of Catalysts

The electrochemical active surface area (ECSA) of the catalysts was evaluated from the following Eq. 6:

6 $$A_{{{\text{ECSA}}}} = \frac{{C_{dl} }}{{40 \mu F cm^{ - 2} }} cm^{2}$$

where *C*_dl_ represents the double layer capacitance and a commonly used specific capacitance (*C*_sp_) value of 40 µF cm^−2^ was used [28, 29].

### Density-Functional Theory (DFT) Calculations

First-principles-based DFT calculations are performed using plane-wave technique implemented in the Vienna Ab initio Simulation Package (VASP) [30]. Projected Augmented Wave (PAW) pseudopotentials are used to define the core electrons [31]. The Generalized Gradient Approximation (GGA) proposed by Perdew, Burke and Ernzerhof is employed to describe electron exchange–correlation interactions [32]. An optimised value of cut-off energy of 450 eV through convergence test is used in the plane-wave basis set. The energy convergence criteria are set to be 10^–5^ eV for electronic self-consistent loop and 10^–4^ eV for ionic relaxation loop. The Brillouin zone sampling within Monkhorst pack scheme is obtained using the 1 × 5 × 1 K-point grid. The vacuum of 20 Å is considered in X and Z directions to avoid the interaction between repeating images.

In nitrogen reduction reaction, the electrochemical activity and reaction mechanism on the catalysts can be estimated by plotting the Free Energy profile. For this, the Gibbs free energies (G) of each reaction steps are calculated by using Eq. 7,

7 $$G = E + {\text{ZPE}}{-}{\text{TS}} - {\text{neU}},$$

where *E* is the DFT energy, *n* is the number of electrons and *U* is the applied potential at the electrode [33], which are provided in the table below for the free molecules like H_2_, NH_3_ and N_2_. (Table 1).

**Table 1:** The DFT energies, entropy terms and zero-point energies for free molecules

Molecule	E (eV)	TS (eV)	ZPE (eV)	G (eV)
H_2_	− 6.77	0.41	0.27	− 6.91
NH_3_	− 19.41	0.58	0.92	− 19.07
N_2_	− 16.19	0.59	0.15	− 16.63

## Results and Discussion

### Energy Optimized Structure and Electronic States of the Catalysts

**Fig. 1:**
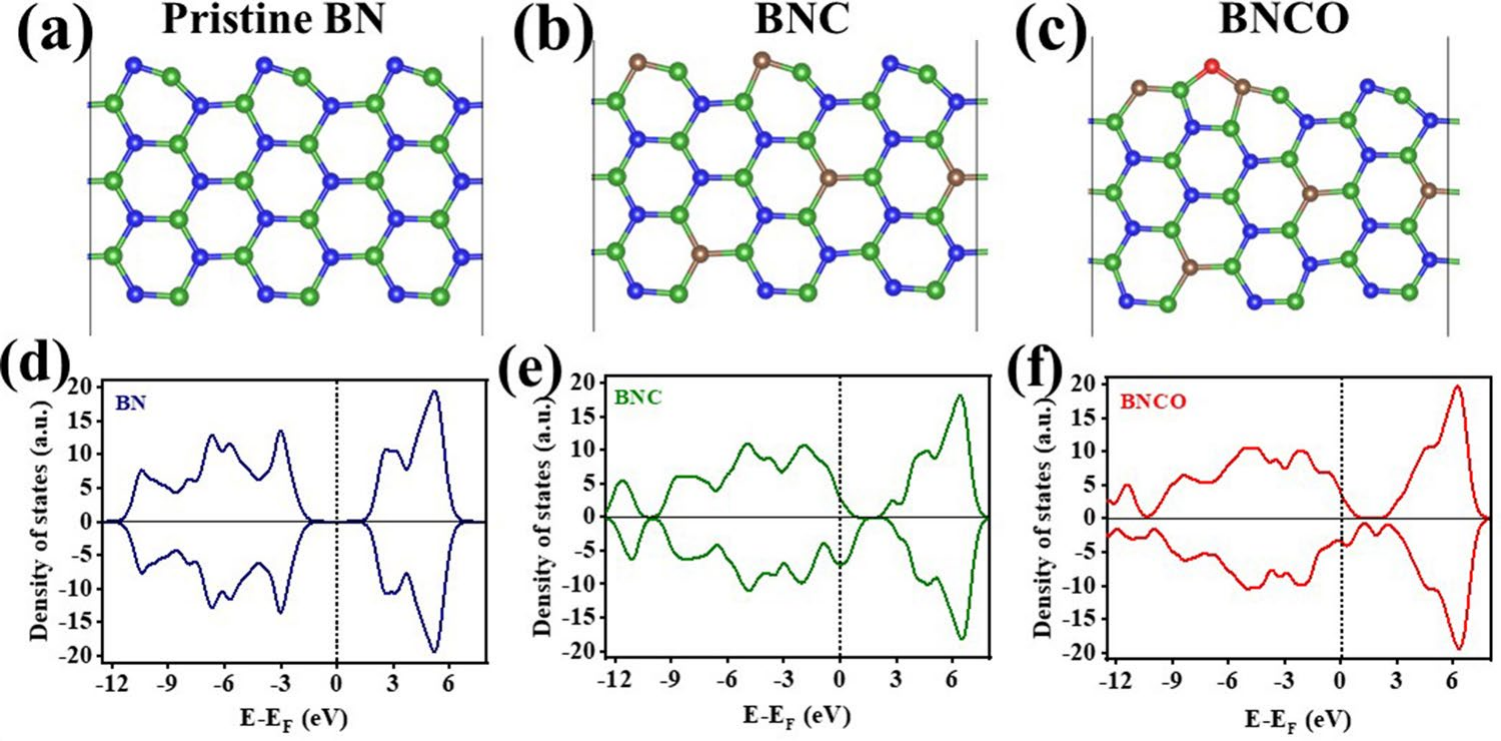
Optimised model structures of **a** BN, **b** BNC, and **c** BNCO systems. The N, C, O, B atoms are denoted with blue, wine, red, green colour spheres, respectively*.* Plot of Density of States for **d** BN, **e** BNC, **f** BNCO systems. The vertical dotted line at zero represents the Fermi Level

Boron atom has gained immense popularity in terms of facile adsorption interaction with N_2_ atoms, owing to the symmetry in their orbital energy. Besides this, B is also known to impart stability to the key intermediates of NRR via a charge balance mechanism [34]. However, in order to make NRR more thermodynamically feasible, a driving force is inevitable, which would account for an enhancement in the charge transfer efficiency of the B active centre to the adsorbed N_2_. The material is expected to become more electronically active, provided the electronic states near its Fermi level is more populated that in turn reinforces the material conductivity [35]. This could be operative in presence of foreign dopants and not only that a better charge density near the Fermi level helps in enhanced N_2_ adsorption and weak binding of the key intermediates of NRR, thereby uplifting the NRR kinetics energetically [36]. In this realm, it was important to realize the correlation between the local charging effect over active B site and its impact on the NRR performance and hence for this work, three energy optimized model structures for pristine BN, BNC, and BNCO were formulated as shown in Fig. 1a-c. Through density of states study, we found that the BNC and BNCO systems became more conductive with large electronic states near Fermi level due to C and O units as compared to BN (Fig. 1d-f) and accordingly we approached to synthesize our desired active material BNCO_(1000)_ along with all the necessary control samples, required to justify the motive of this work.

### Structural Illustration and Formation Mechanism of the Catalyst

**Fig. 2:**
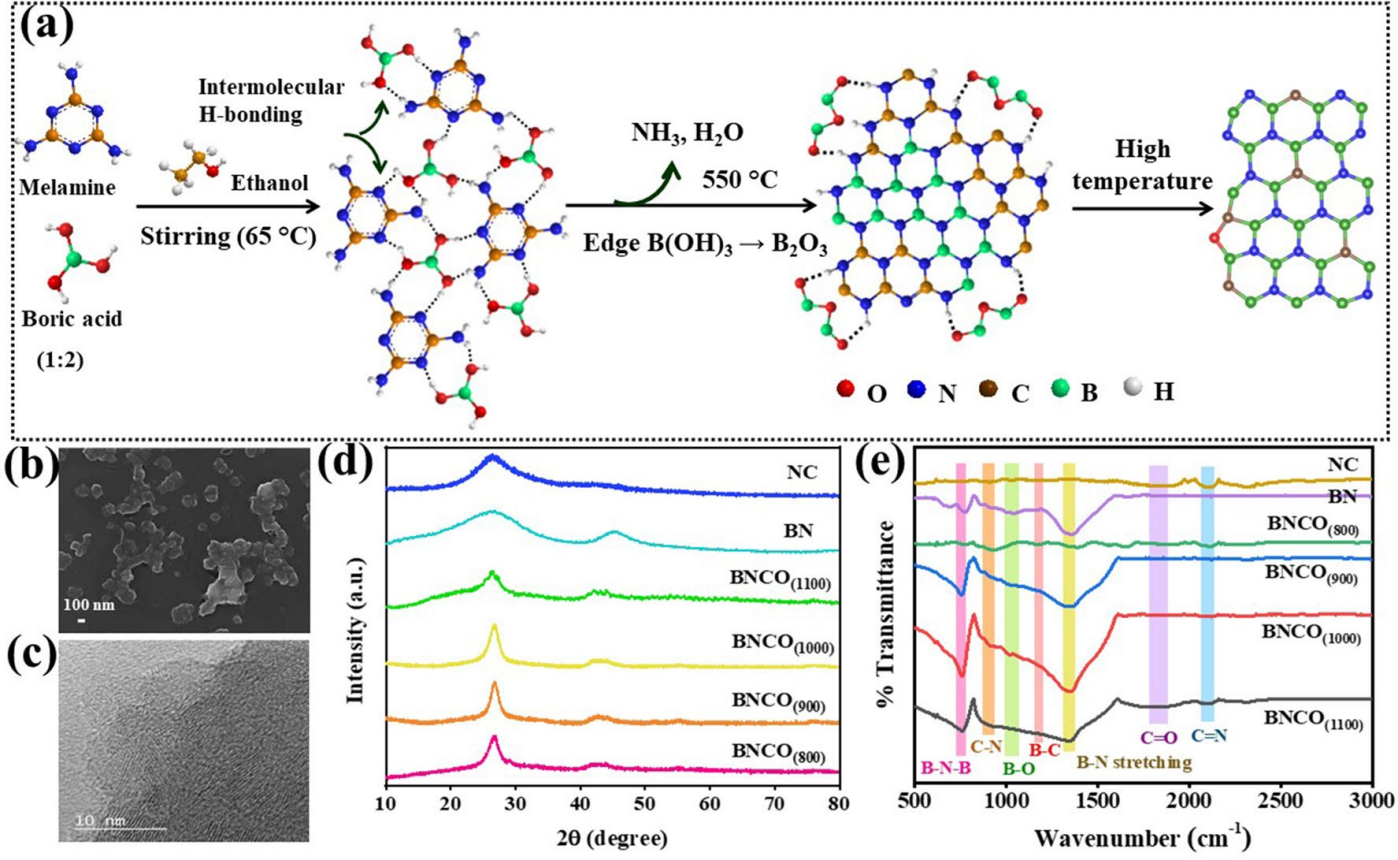
**a** Schematic representation of the probable mechanism of BNCO_(1000)_ catalyst synthesis by pyrolysis method. **b** FESEM image of BNCO_(1000)_ catalyst (scale-bar: 100 nm). **c** High resolution TEM image of BNCO_(1000)_ catalyst (scale bar: 10 nm). **d** XRD of all the synthesized catalysts. **e** FTIR spectra of all the as-synthesized catalysts

The synthesis of the active material initiated with mechanical stirring of the precursors, melamine and boric acid at 65 °C in ethanol, where an inevitable H-bonding between the two moieties enable the formation of a single-sourced co-crystal for the synthesis of boron carbonitride framework [37]. Nevertheless, it is important to mention the significance of the two-step pyrolysis process towards NRR activity of the material. At 550 °C, a condensation reaction would eliminate H_2_O and NH_3_ from the precursor and an irregular architecture of boron nitride and carbon-nitride is expected to form. However, as boric acid is taken in excess, there persists a possibility of further inter-molecular H-bonding at the edges as depicted in Fig. 2a. Beyond 550 °C, the B(OH)_3_ units get converted to B_2_O_3_, which further take part in the high temperature pyrolysis step to deliver our final active catalyst, oxygen edge-functionalized boron-carbonitride (BNCO_(1000)_). So, the structural disintegration followed by elemental re-organization of the precursors led to the formation of boron carbonitride architecture with well-defined boron active sites per hexagon unit and islands of edge pentagons containing O functionality. The synthesis method and the probable mechanism has been schematically elaborated in Fig. 2a.

### Morphology and Structural Characterization

**Fig. 3:**
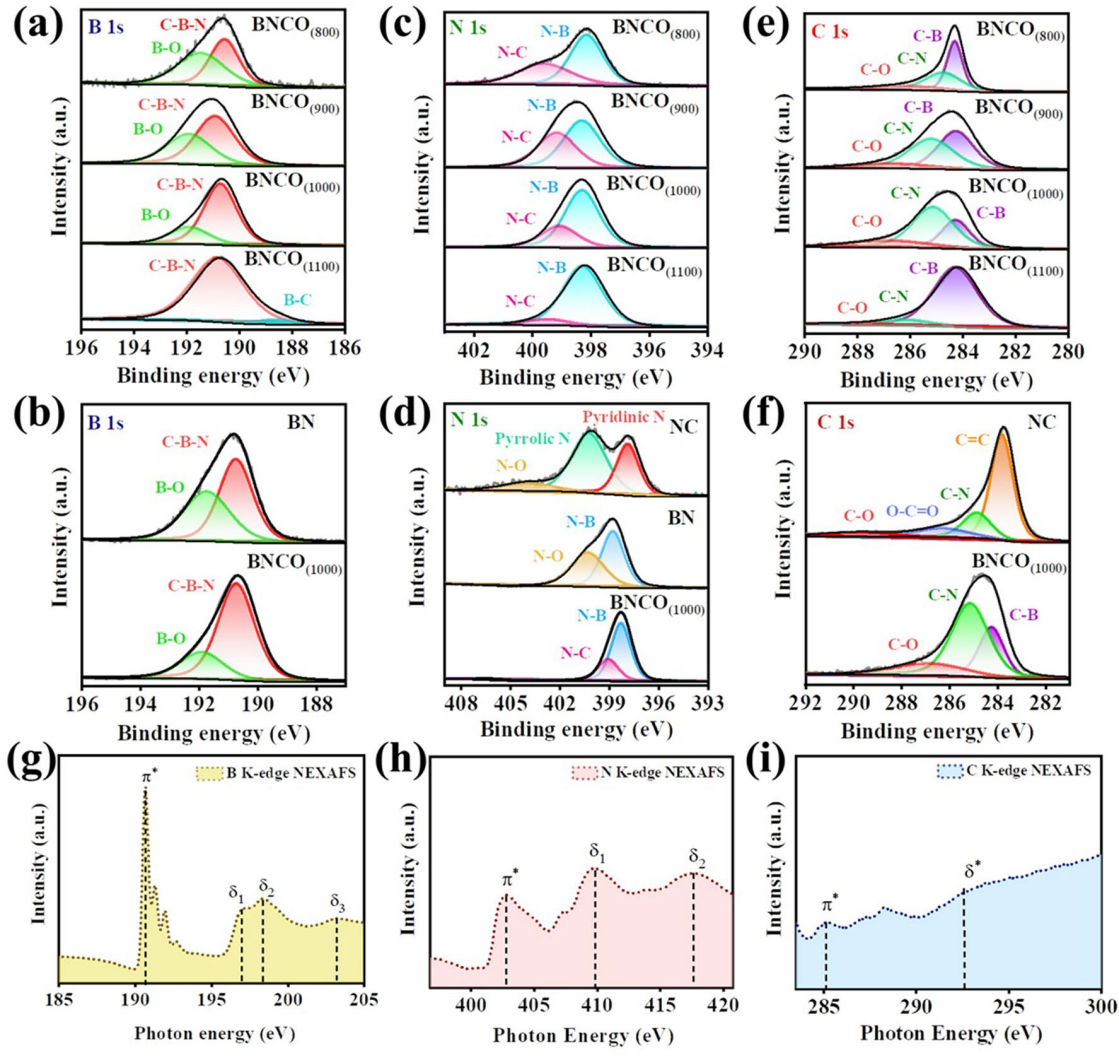
Comparative narrow XPS spectra of all the catalysts at **a, b** B 1*s*, **c, d** N 1*s*, **e, f** C 1*s* edges. NEXAFS spectra of BNCO_(1000)_ catalyst at **g** B *K*-edge, **h** N *K*-edge, and **i** C *K*-edge

The field emission scanning electron microscopy (FESEM) image in *G* = *E* + ZPE–TS-neU, Fig. 2b presents that the catalyst resembled a hardened granular morphology, which could be attributed to the crosslinking effect of the intramolecular boronic acid and melem moieties forming the BNCO_(1000)_ active material [38]. The irregularly organized lattice fringes in the high-resolution transmission electron microscopy (HRTEM) image in Fig. 2c depicted the crystalline nature of the material that could be also evident from the selected area electron diffraction (SAED) pattern (Fig. S1). The elemental composition of the BNCO_(1000)_ catalyst was verified by means of FESEM mapping, where Fig. S2 evidences the presence of B, N, C, and O atoms. The X-ray diffraction (XRD) pattern of all the synthesized materials was consistent with that obtained for C-doped BN type of material architecture (Fig. 2d) [39]. The characteristic peaks located at 2θ of 26 and 43° could be ascribed to the (002) and (100) planes of BCN [40–42]. The slight shifting in the (100) plane to a lower angle from the XRD spectrum of pristine BN originated from the planar strains that could have developed due to the C doping and O edge-functionalization [43, 44]. This necessitated the establishment of the elemental bonds constituting the catalysts, which was verified from Fourier-transform infrared (FTIR) spectroscopy. As demonstrated in several reports, the formation of BN architecture occurs at a temperature of 900 °C and above, the nature of the FTIR spectra for the catalysts BNCO_(900)_, BNCO_(1000)_, and BNCO_(1100)_ was consistent with that obtained for BN with the signature vibrations appearing at 1345 and 761 cm^−1^ for B–N stretching and B–N–B bond, respectively, as shown in Fig. 2e [39, 45, 46]. The other peaks corresponding to B–C, C–N [47], B–O–C, C=O, and C=N bonds could also be seen from the stretches in between 900 and 1400, ~ 1800, and 2100 cm^−1^, respectively. The chemical bonding environment within the BNCO_(1000)_ catalyst was further determined by X-ray photoelectron spectroscopy (XPS). A comparative XPS study of all the catalysts has been provided to have a clear vision of the formation of our active catalyst. The survey spectra in Fig. S3 showed the presence of all the corresponding elements B, C, N, and O in the as-prepared samples. The high resolution B 1*s* spectra in Fig. 3a evidence the gradual formation of the C-doped BN structure with C–B–N peak at 190.73 eV [48], along with the lowering in the integral peak area of B–O (at 192 eV) [18, 46] with the gradual rise in temperature from 800 to 1000 °C. At 1100 °C, the B–O peak disappeared along with the emergence of a small peak at 188 eV corresponding to B–C bond. The deconvoluted peaks appeared identical for the BNCO_(1000)_ as well as pristine BN samples in Fig. 3b, where the peak corresponding to B–O could be reasonably from the O edge-functionalization of the B centres. Likewise, in the N 1*s* narrow spectra in Fig. 3c, the peak corresponding to N–C at 399.58 eV appeared to be rather broadened in the sample formed at 800 °C along with the presence of N-B bonding characteristic at 398.15 eV. It could be expected that at this temperature, a disordered structure was formed which eventually got reorganized at a temperature of 900 to 1100 °C, where a gradual increment in the N–B peak intensity could be observed with a simultaneous lowering of the N–C peak intensity. The greater number of B–N pairs manifested greater number of active sites at 1000 °C, beneficial for NRR [18]. However, although it appeared that the N–B peak got even more intense in case of 1100 °C, the relative elemental content of this material BNCO_(1100)_ revealed a lowering in the B and N atomic percentage with simultaneous rise in the C content of the material as tabulated in Table S1. In case of pristine NC, pyridinic N, pyrrolic N and N-oxides could be seen from the peaks at 397.9, 400.3, and 403.9 eV, respectively, in Fig. 3d [49], while for pristine BN, peaks corresponding to N–B and N–O could be evidenced at 398.8 and 400.2 eV, respectively. The N–B peak in the final material was found to be shifted to a lower binding energy by 0.6 eV, which could be attributed to the disorderness in the structure due to foreign dopants like C and O. In the high-resolution C 1*s* spectra in Fig. 3e, a sharp fall in the C–B peak intensity could be seen with rise in pyrolysis temperature from 800 to 1000 °C, which was an indication for the formation of more BN motifs with repetitive C doping per hexagonal unit of BN, consistent with that obtained in N 1*s* spectrum [41]. But due to rise in the C content with further rise in temperature to 1100 °C, assumingly there occurred a structural disruption from the regular C-doped BN framework and an increase in the extent of graphitization enhanced the C content in the material with emergence of a broad C–B bond in the C 1*s* spectrum. In the B 1*s* spectrum, for 1100 °C material, there was appearance of a new peak corresponding to B–C, besides the conventional C–B–N peak, corroborating our finding from the C 1*s* spectra. However, the appearance of C–O peak also indicated the presence of O functionalization at the edges of all the synthesized catalysts (Fig. 3e-f). It is not surprising that the high resolution O 1*s* spectra in Fig. S4 disclosed the same conclusion drawn from the B 1*s* and C 1*s* spectra regarding the B–O and C–O bonds, though it was found that the O content gradually diminished at higher pyrolysis temperature (Table S1).

In order to have some insightful idea about the local electronic structures of the BNCO_(1000)_ material, near-edge X-ray absorption fine structure spectroscopy (NEXAFS) was employed. Basically, in NEXFAS technique, a core level electron is excited to a partially unoccupied higher orbital level and hence the spectra obtained from NEXAFS is directly associated with the nature of the unoccupied orbital states. The B 1*s* NEXAFS (Fig. 3g) spectrum showed a characteristic sharp peak at 190.67 eV for the B 1*s* π* transition, which could be designated to the *sp*^2^ hybridized, planar edge B atoms [50]. The three resonances at 197.02, 198.48, and 203.2 eV (represented as δ_1_, δ_2_, and δ_3_) were attributed to the σ* excitations, where the former two involved antibonding interactions between N 2*s* and B 2*p*_xy_ orbitals, while the later represented second harmonic interaction between N 2*p*_xy_ and B 2*p*_xy_ orbitals [51]. These stretches resembled that of *h*-BN [52]. The shoulder peaks at ~ 192.6 eV were attributed to the B–O motifs, possibly originating from the unreacted boron oxides, as complied with the XPS narrow spectrum for B 1*s* [38]. The traces of B–O units were also evident from the O 1*s* NEXAFS as shown in Fig. S5 [53]. In all of the cases, a negative shift could be observed in the binding energies for our material BNCO_(1000)_ than that reported for pristine h-BN, which was due to some tensile strain due to the C doping effect, that affected the intraplanar local structure. For the N 1*s* NEXAFS in Fig. 3h, the peak at 402.8 eV denoted the π* resonance while the broadened peaks at 409.7 eV (δ_1_) and 417.6 eV (δ_2_) were attributed to the σ* features and the formation of C–B–N bond [54]. The slight shift in the π* peak position was due to the fact that N, being more electronegative imposed a more polarization effect on the B–N bond in BNCO_(1000)_ system that likely impacted the binding energies of the participating bonding electrons [52, 55]. The C 1*s* NEXAFS spectrum in Fig. 3i evidenced the presence of two distinguishable peaks at 285.2 (π*) and 292.6 (δ*) eV for the 1*s* to π* and 1*s* to σ* resonances of the *sp*^2^ hybridized C, respectively [56]. All of the above-mentioned characterizations provide ample evidence for the formation of distinct C-doped BN moiety with O functionalization at the edges, where BNCO_(1000)_ catalyst serves as a potent candidate for NRR. The electronegativity of O and the charging effect between C–B–N unit combats the potential intensive steps of NRR and bring about facile ammonia production as discussed in the later section.

### Electrochemical NRR Performance: Role of Electrolyte Anions and B-active Units for Improved NRR Kinetics

**Fig. 4:**
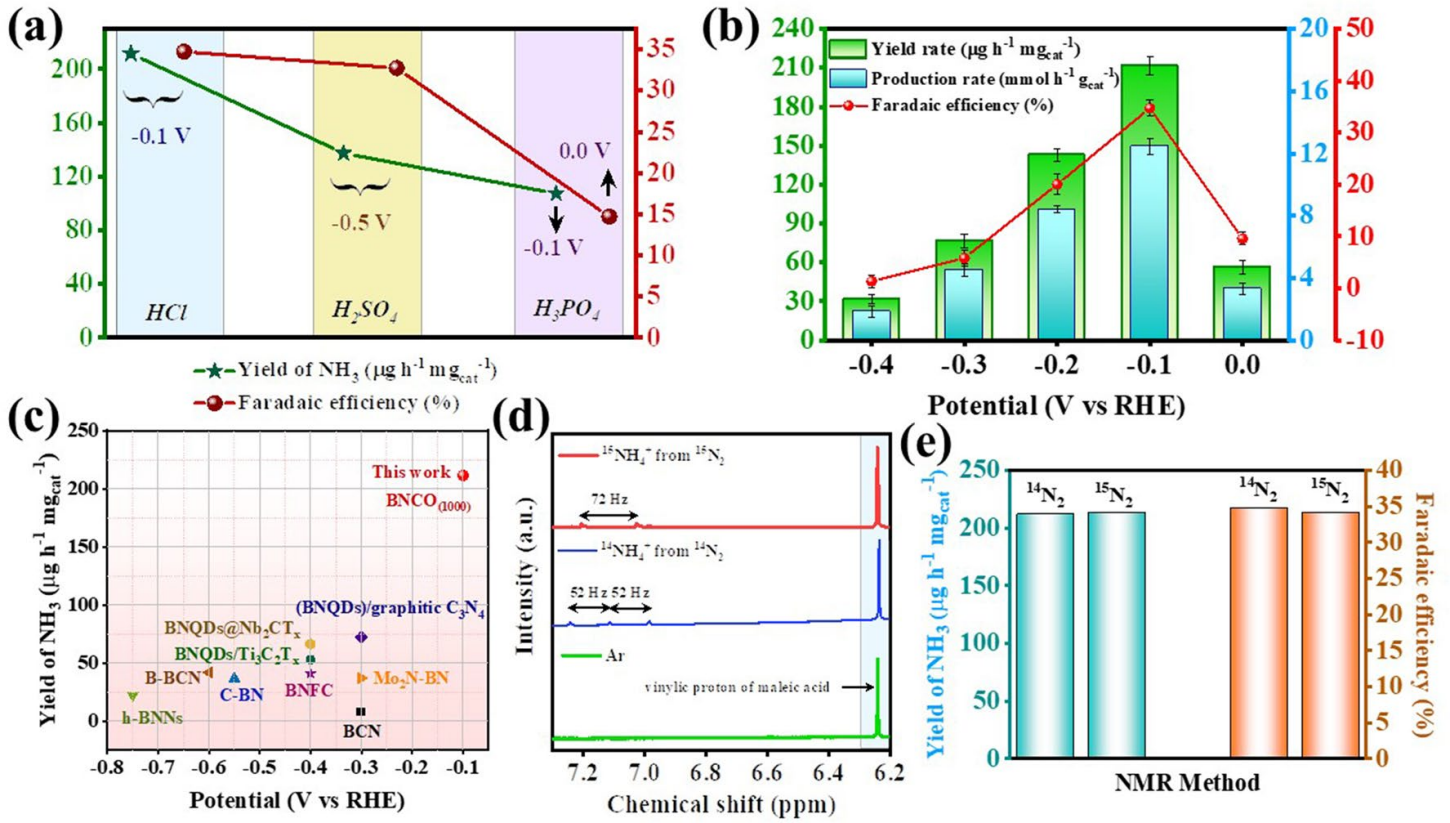
**a** NRR performance of BNCO_(1000)_ catalyst in different electrolyte conditions (0.1 M HCl, 0.1 M H_2_SO_4_ and 0.1 M H_3_PO_4_). **b** Comparative plot showing yield and FE of NH_3_ synthesis over the wide-ranging potential window for BNCO_(1000)_ catalyst in 0.1 M HCl. **c** Yield of ammonia with respect to potential of our work, compared with all the BN or BNC class of catalysts reported so far for NRR. **d** Isotope labelling experiment displaying ^1^H NMR spectra obtained after NRR in 0.1 M HCl with Ar, ^14^N_2_ and ^15^N_2_ feeding gases. **e** Comparison of ammonia yield and Faradaic efficiency, quantified by NMR method with ^14^N_2_ and ^15^N_2_ feeding gases with BNCO_(1000)_ catalyst

The lone pair of electrons over nitrogen brings about a weak Lewis basicity in the N_2_ molecules, while the Lewis acidic nature of the B atom makes it a suitable candidate for N_2_ adsorption [18]. In order to facilitate the protonation of the adsorbed N_2_ molecules, it is necessary to maintain a proton sufficiency in the medium. This makes the use of acidic electrolyte an optimum choice for NRR [57–59]. In fact, acidic electrolyte acts as a proper trap to capture all the converted ammonia in form of NH_4_^+^. Thus, for this work, primarily three acidic electrolytes were considered for NRR that, is, 0.1 M HCl, 0.1 M H_2_SO_4_ and 0.1 M H_3_PO_4_. The comparative linear sweep voltammetry (LSV) curves of the active material BNCO_(1000)_ in N_2_ purged condition (Fig. S6) displayed a better NRR onset potential for H_3_PO_4_, though performance-wise HCl stood as a better competent. A fair explanation to this could be the better adsorption and binding probability of N_2_ by phosphate radicals (PO_4_^3−^-N_2_), which increases the N–N bond length to a greater extent as compared to SO_4_^2−^ and Cl^−^, as shown in Fig. S7. Moreover, from chemistry point of view, P and N being same group elements, their 3*p*-2*p* overlap leads to an enhanced interaction which helps to bring N_2_ into the solid–liquid-gas interface and initiate NRR at a lower overpotential. The NRR activity was also reasonably good for both H_3_PO_4_ and H_2_SO_4_ as obtained from the chronoamperometric scans over a wide-ranging potential window and verified from UV–visible spectra of the colorimetric detection of NH_3_ (Indophenol-blue method) in Figs. S8 and S9. But considering the proton donating ability of HCl, a stronger acid than H_2_SO_4_ and H_3_PO_4_, the NRR performance exceeded the latter two as shown in Figs. S10 and 4a. More importantly, the bulky anions SO_4_^2−^ and PO_4_^3−^ led to a crowding effect and blocked the B active sites by binding with B through O end (Lewis acid–base interaction). The thermodynamic favourability of this phenomenon as shown in Fig. S11 hindered the smooth pathway for N_2_ adsorption over the B active sites in case of H_2_SO_4_ and H_3_PO_4_. The theoretical results revealed that a greater charging interaction was induced from B to PO_4_ than SO_4_ and is the lowest for Cl anion in Fig. S12, which accorded with the NRR activity trend found experimentally (Fig. 4a). Table S2 provides a summary of the NRR performance of BNCO_(1000)_ catalyst in the three above-mentioned electrolytes. However, it is important to secure the B active centres in order to get an uninterrupted NRR activity. Thus, 0.1 M HCl was considered to have an optimum acidity and anion effect to persuade facile NRR and was taken as the working electrolyte for all the electrochemical studies. It is noteworthy to mention that in all the above cases, there was no perturbation from side product formation (hydrazine) as shown in Fig. S13. In all cases, the concentration of NH_3_ and N_2_H_4_ (side product) was calculated from the UV–visible calibration curves obtained from the respective Indophenol-blue and Watt and Chrisp methods (Figs. S14-S17). Nevertheless, for an elaborate NRR study of our active material in 0.1 M HCl, the LSV polarization curves were produced at 10 mV s^−1^ scan rate in an ideal three-electrode system, where a notable difference in current densities could be observed in Ar and N_2_ fed electrolyte conditions (Fig. S18). It is important to mention that all the potentials referred in this work are calibrated with respect to reversible hydrogen electrode (RHE), following Eq. 1. Considering the working potential range from 0 to − 0.4 V in case of 0.1 M HCl, potential-dependent scans were taken, holding each potential for 2 h time. It was observed in Fig. 4b that the highest yield of ammonia was obtained to be 211.5 μg h^−1^ mg_cat_^−1^ at − 0.1 V with a *FE* of 34.7% and 12.44 mmol h^−1^ g_cat_^−1^ mass-normalized ammonia production rate, calculated from Eqs. 2–4. According to the best of our perception, this is the highest reported yield of ammonia on boron nitride or boron-carbonitride class of materials (Fig. 4c), where B actively served as the unit for N_2_ adsorption and subsequent reduction as shown in Table S3 [17, 18, 29, 51, 60–64]. The high production of ammonia was further verified from the isotope labelling experiment, where distinguishable triplet and doublet ^1^H NMR signals with coupling constant values of 52 and 72 Hz, respectively, were evident for ^14^NH_4_^+^ and ^15^NH_4_^+^, while there was no signal in case of Ar saturated condition of electrocatalysis (Fig. 4d). More convincing evidence to the production of ammonia was provided by the quantitative analysis with maleic acid using Eq. 5, where from the yield and Faradaic efficiency of ammonia was calculated and cross verified to be almost similar with both ^14^N_2_ and ^15^N_2_ feeding gases (Figs. S19 and 4e).

**Fig. 5:**
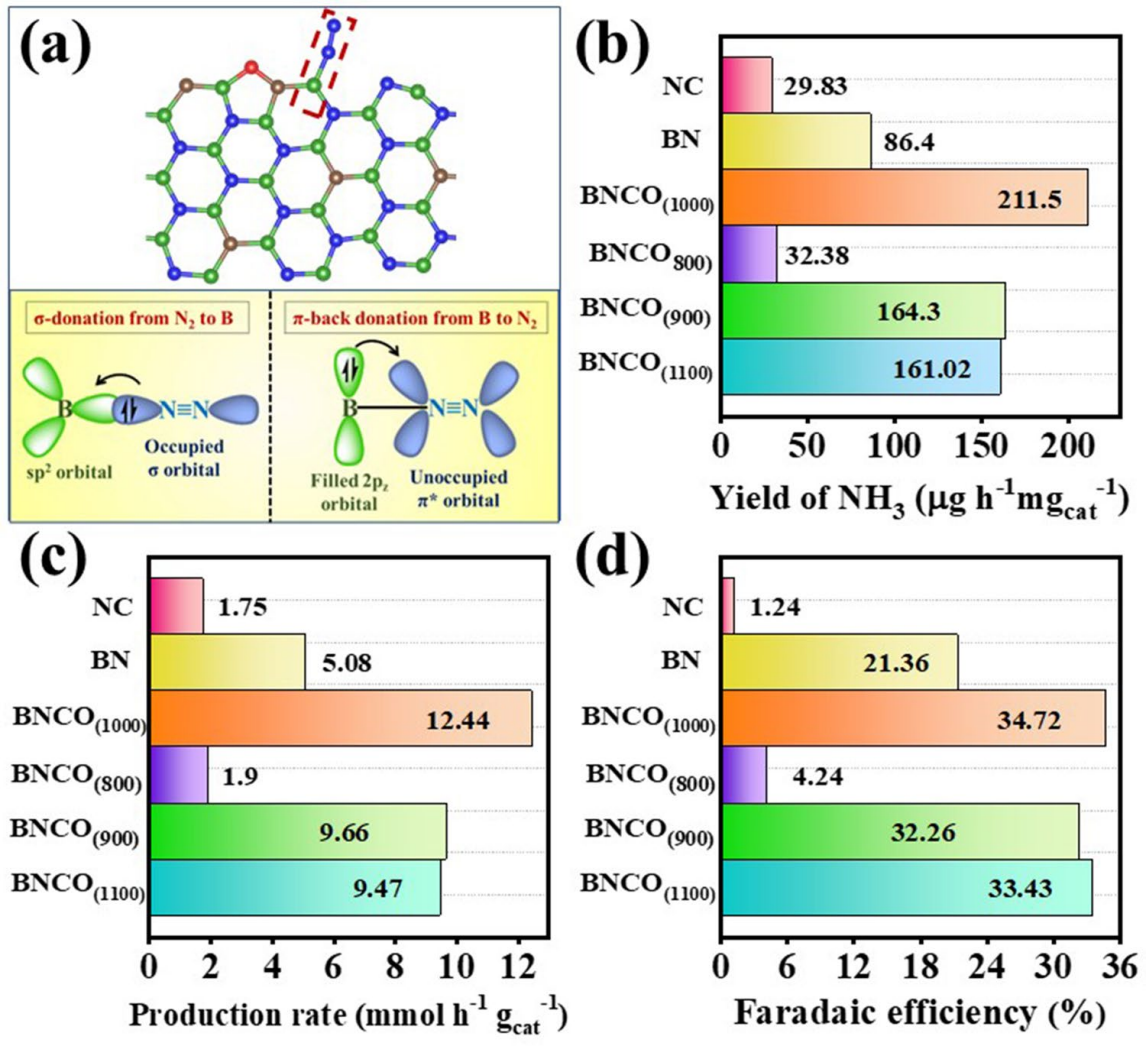
**a** Energy optimized structures of the BNCO_(1000)_ catalyst with N_2_ adsorbed on the edge B site and the molecular orbital interaction between B and ad-N_2_. Comparison in NRR performance of all the synthesized catalysts in 0.1 M HCl at -0.1 V vs RHE in terms of **b** yield of NH_3_, **c** mass-normalized production rate and **d** Faradaic efficiency

Boron is well known to have a proper orbital compatibility with N_2_ that plays a significant role to lower down the free energy requirement for N_2_ adsorption [65]. Particularly, the B atoms at the edges with were found to be active for N_2_ adsorption [16]. As schematically shown in Fig. 5a, the unoccupied *sp*^2^ hybrid orbital of B took away the electron density from the highest occupied molecular orbital- σ (HOMO) of N_2_ and in turn donated the π-electron cloud into the lowest unoccupied molecular orbital-π* (LUMO) of N_2_ from the filled 2*p*_z_ orbital of B via back-bonding. This interaction induced the N≡N bond weakening and brought about facile first protonation, which is the so-called “potential determining step” of NRR on B active sites. While the role of B active unit was evident from the insignificant NRR performance in NC with NH_3_ yield to be 29.83 μg h^−1^ mg_cat_^−1^, the presence of C had profound role in proliferating the NH_3_ yield rate and Faradaic efficiency than pristine BN (Fig. 5b-d). Interestingly, as proper BN architecture was expected to form at a temperature of 900 °C and above, the BNCO catalysts (BNCO_(900)_ and BNCO_(1000)_) formed at this high pyrolysis temperature displayed better performance (NH_3_ yield for BNCO_(900)_ 164.3 μg h^−1^ mg_cat_^−1^, while for BNCO_(1000),_ the yield was 211.5 μg h^−1^ mg_cat_^−1^) as compared to BN and BNCO_(800)_ (NH_3_ yield for BN 86.4 μg h^−1^ mg_cat_^−1^, while for BNCO_(800),_ the yield was found to be 32.38 μg h^−1^ mg_cat_^−1^). However, the catalyst synthesized at 1100 °C (BNCO_(1100)_) slightly lagged in performance, which could be attributed to the loss of B active sites as shown from the lowered B content in the relative atomic % of the material from XPS analysis (NH_3_ yield for BNCO_(1100)_ 161.02 μg h^−1^ mg_cat_^−1^). The performances of all the catalysts in terms of current density, NRR onset potential, ammonia yield, production rate and Faradaic efficiency are summarized in Figs. S20-S22, 5b-d and Table S4. Blessing of the optimized synthesis conditions, our final material BNCO_(1000)_ yielded 211.5 μg h^−1^ mg_cat_^−1^ ammonia synthesis with 34.7% Faradaic efficiency. The electrochemical active surface area (ECSA) had a direct co-relation with the electrocatalytic activity, and the results obtained for all our catalysts were absolutely in congruence with the NRR performances of the materials. The ECSA of the catalysts were evaluated by taking cyclic voltammetry (CV) at varied scan rates as shown in Fig. S23a-f. A plot of Δ*j* = (*j*_a_ – *j*_c_) against the scan rate was linearly fitted in order to obtain the slope corresponding to twice the double layer capacitance (*C*_dl_) of the materials (Fig. S24). Thereafter using Eq. 6 and as shown in Table S5, the maximum ECSA was obtained for BNCO_(1000)_, owing to which it displayed maximum potency towards NRR.

### Mechanism of the Active Material Towards NRR from DFT Point of View

**Fig. 6:**
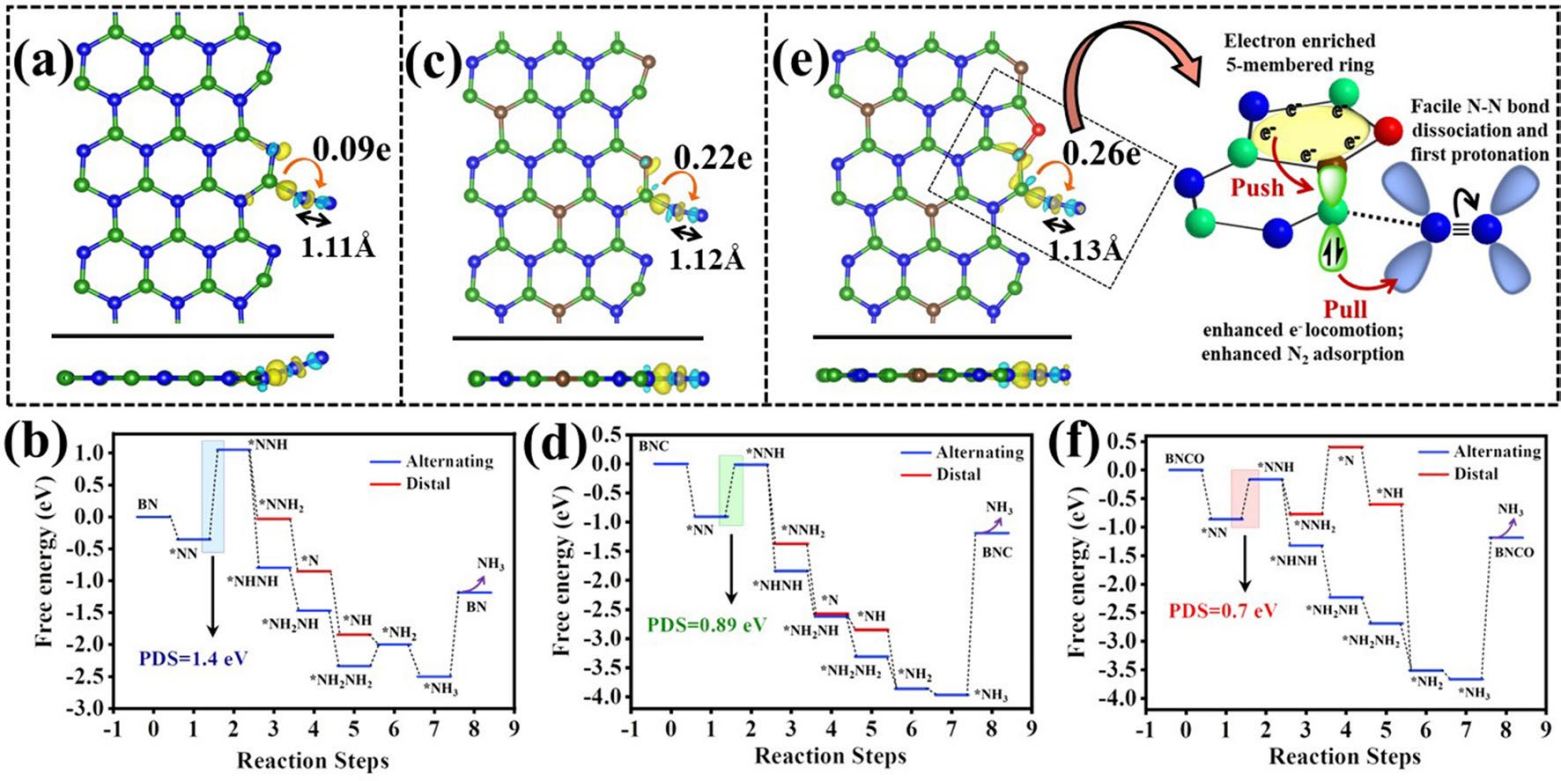
Charge density difference analysis for **a** pristine BN, **c** BNC, and **e** BNCO systems. Yellow and blue lobes indicate electron accumulation and depletion, respectively (Iso-surface value = 0.001 e Å^−3^). The arrow and value indicate amount of Bader charge transferred. Free energy profile for NRR for **b** BN, **d** BNC, **f** BNCO

Theoretical analysis was carried out to unveil the role of carbon and oxygen towards improvement in NRR activity in BN systems. It is known that boron site is more promising active site to adsorb N_2_ for eNRR as compared to nitrogen sites [16]. Therefore, we considered the single boron site at the edge (adjacent to the CO group in Fig. 1c) in all three models to investigate the NRR mechanism. Firstly, we performed the N_2_ adsorption study on boron site of these three models. We found that the end-on configuration of N_2_ adsorption is more favourable with lower free energy (Eq. 7) than side-on configuration on boron site [16]. Therefore, the alternating and distal pathways could only occur towards NRR, whereas the enzymatic or mixed mechanisms were not feasible. Despite the importance of B in NRR, the pivot of this work was to reinforce the local charging effect over B to facilitate the charge transportation from B to N_2_, which was successfully accomplished by the presence of foreign dopants like C and O. Through charge density difference analysis and Bader charge study, the adsorption of N_2_ on boron site in various models (Fig. S25) were investigated. In case of pristine BN in Fig. 6a-b, a lowered charge transfer from B to N_2_ was evidenced (0.09e), which in turn resulted in a lower N≡N bond cleavage tendency and greater overpotential of PDS (ΔG_N2_ – ΔG_NNH_ = 1.4 eV) for NRR. In the full free energy profile, we observed the lower value of free energy of NHNH step compare to the NNH_2_ step that prefers the alternating pathway over the distal pathway [66–68]. Further, the systems BNC and BNCO also showed a same PDS (ΔG_N2_ – ΔG_NNH_) and alternating pathway for NRR. However, in the presence of carbon, the edge C atoms had a propagating effect on the B active units that resulted in enhanced charge transfer (from 0.09e in case of pristine BN to 0.22e in BCN) from B to N_2_, reinforcing N_2_ adsorption at a much-reduced overpotential (from 1.4 to 0.89 eV) (Fig. 6c-d). Furthermore, edge-functionalized oxygen atom with carbon atoms formed the stable pentagon ring that made the carbon-boron bond weaker and the boron site became more active to adsorb N_2_ strongly. Therefore, this pentagon ring behaved as an electron reservoir to enrich the local electron density over B such that an enhancement of charge transfer (0.26e) could be evident from B to adsorbed N_2_ with lowering of the N_2_ reduction overpotential to 0.7 eV (Fig. 6e-f). The N_2_ activation is dependent on its bond length after adsorption, where BNCO system shows enhanced N_2_ activation due to higher bond length of adsorbed N_2_ as follows, 1.13 Å (BNCO) > 1.12 Å (BNC) > 1.11 Å (BN). Thus, the charging effect and importance of O functionalization along with C dopants could be established for the emerging BN class of materials for NRR.

To understand the effect HER on NRR theoretically, we performed the hydrogen adsorption calculation on the boron active site of BNCO system. In the comparison of free energy profile, we find that the NRR is more dominant on HER due to two reasons, (i) Higher N_2_ adsorption energy (− 0.86 eV) than hydrogen adsorption (− 0.77 eV) on boron site, (ii) Lower value of NRR overpotential (0.7 V) than HER overpotential (0.77 V) (Fig. S26). Therefore, the BNCO catalyst is more favourable for NRR by supressing competitive HER.

### Cyclability and Stability Studies of BNCO_(1000)_ Catalyst

**Fig. 7:**
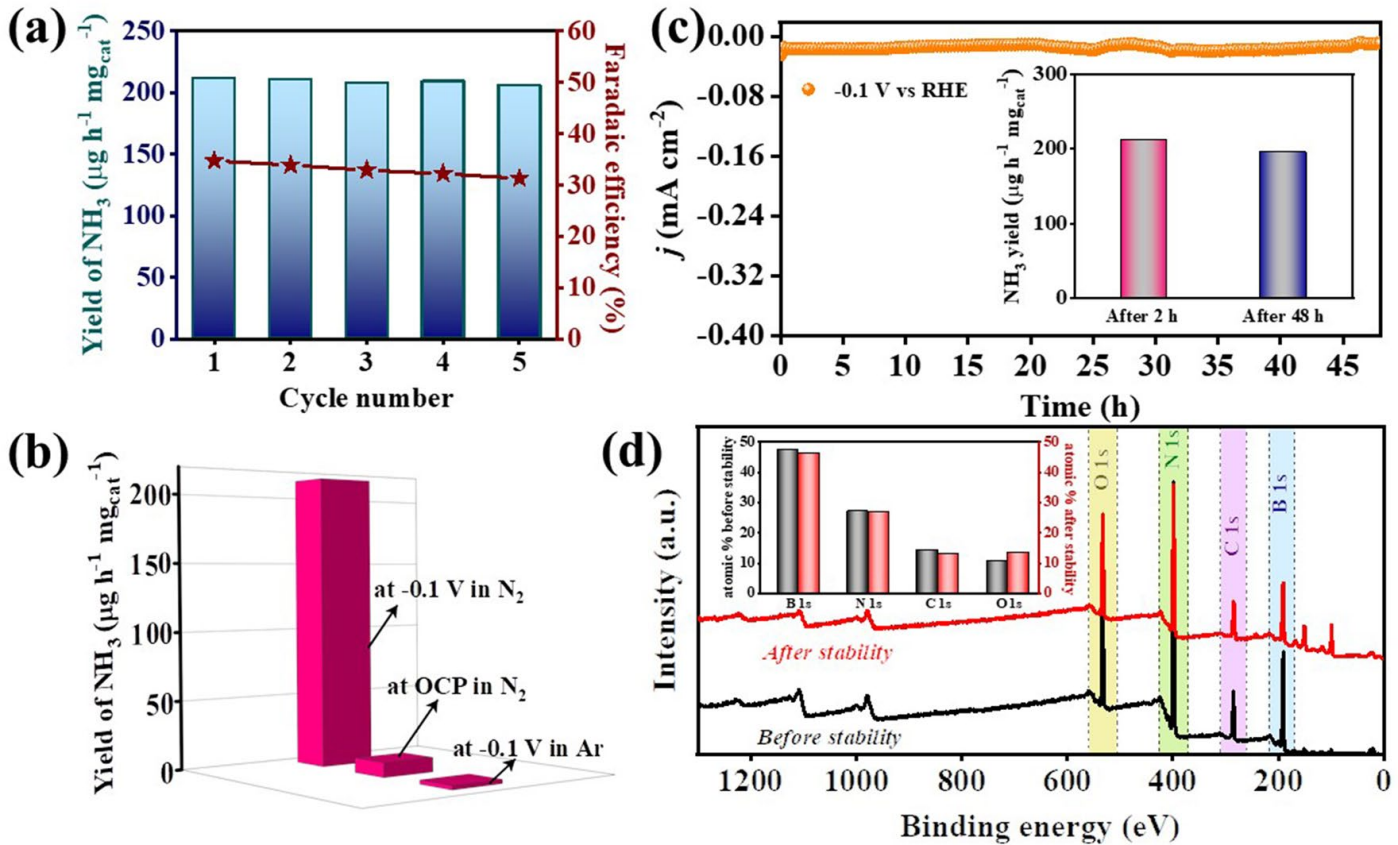
**a** Yield of NH_3_ and FE of ammonia production on BNCO_(1000)_ catalyst after cycling experiments, holding − 0.1 V vs RHE for 2 h for each cycle. **b** Comparative bar plot of yield of NH_3_ in N_2_ and Ar, at − 0.1 V and in N_2_ at OCP for BNCO_(1000)_ catalyst (2 h chronoamperometric test). **c** Stability of BNCO_(1000)_ catalyst for 48 h at − 0.1 V, inset showing NH_3_ yield after 2 h and 48 h. **d** Before and after stability comparison of XPS full survey spectra of BNCO_(1000)_ catalyst, inset shows the bar diagram of the atomic % of B, N, C, and O for fresh and after-stability samples

The viability of the catalyst (BNCO_(1000)_) performance was ensured from repetitive cyclability measurements, holding − 0.1 V vs RHE for 2 h, per cycle (Fig. S27a). It was interesting to found that the overlying UV–visible spectra in Fig. S27b produced similar concentration of ammonia. The post-cycling ^11^B and ^13^C NMR studies for the electrolyte (0.1 M HCl) were performed to verify whether the catalyst was dissolved in the medium in due course of the reaction. Keeping in mind the natural abundance of these isotopes and the concentration of material that could have dissolved in the electrolyte, 8000 scans and 6000 scans were applied prior to ^13^C and ^11^B NMR measurements, respectively. As can be seen from Fig. S28a, the ^13^C NMR spectrum displayed only one peak corresponding to the solvent DMSO-d^6^. There was no peak corresponding to the material (BNCO_(1000)_) either in ^13^C or ^11^B (Fig. S28b) NMR data, which certainly ensured that the material was not soluble in the electrolyte and the partial deactivation of the active site could be possibly responsible for the minimal drop in Faradaic efficiency, while there was a harmony in the yield of NH_3_ produced during each of the five chronoamperometric cycles (Fig. 7a). Besides, the isotope labelling experiment, several control experiments like (a) At − 0.1 V vs RHE in Ar purged condition and (b) At open circuit potential (OCP) in N_2_ purged electrolyte (Fig. S29), negligible yield of ammonia further confirmed the authenticity of ammonia production, chiefly from the feeding gas (Fig. 7b). In case of N-containing samples, it becomes imperative to show that the catalyst N had no interference in the conversion to N to ammonia. Although, from isotope labelling experiment, the source of the ammonia obtained in the catholyte was verified to be from the feeding gas, a prolonged stability experiment was run to further check the stability and efficiency of the catalyst. After a chronoamperometric run of 48 h at − 0.1 V vs RHE under continuous N_2_ purging (Fig. 7c), there was only a trivial loss in the yield rate of ammonia as evident from the inset of Fig. 7c. This proved that the catalyst was efficient enough to be used for a prolonged time in the N_2_ purged electrolyte condition under a continuous − 0.1 V potential. The after stability FTIR spectra (Fig. S30) of the catalyst displayed identical stretching vibrations of the elemental bonds present in the BNCO_(1000)_ material as that in the fresh sample. In fact, XPS full survey spectra of the material revealed similar percentage of elemental content as the fresh sample, particularly that of N (Fig. 7d, inset shows the bar diagram of the elemental% of fresh and used material BNCO_(1000)_). More vividly, the narrow spectrum of B 1*s*, C 1*s*, and N 1*s* also showed similar integral areas of the deconvoluted peaks as shown in Fig. S31a-c and Table S6. Thus, this material proved to possess enough potential to be used as a metal-free electrocatalyst with target-specific, charge polarized and electrolyte-secured edge B active centres for NRR in 0.1 M HCl.

## Conclusions

In summary, this work displayed the significant role of O and C doping within BN architecture to promote NRR on the edge B sites via associative alternating mechanism. The gradual formation of the ideal structure was systematically studied by means of XPS and the electronic properties were investigated from NEXAFS. A greater impact was found on the charging effect of B centres due to O-functionalized edges that induced a greater charge density from B to the adsorbed N_2_, overcoming the potential determining steps for NRR. This work simultaneously highlighted the importance of choice of electrolyte, where in 0.1 M HCl our catalyst BNCO_(1000)_ yielded 211.5 μg h^−1^ mg_cat_^−1^ of ammonia at − 0.1 V vs RHE with a FE of 34.7%. It was experimentally found and theoretically supported that the bulky anions in H_2_SO_4_ and H_3_PO_4_ blocked the B active sites by a Lewis acid–base interaction between the B sites and the O ends of the anions, hence not suitable for this class of materials. Thus, our present work offered an overall idea of catalyst designing and the importance of medium to retain a high and consistent NRR performance.

## Supplementary Information

Below is the link to the electronic supplementary material.

Supplementary file1 (PDF 2350 KB)

## References

[1] Ren Y, Yu C, Tan X, Huang H, Wei Q (2021). Strategies to suppress hydrogen evolution for highly selective electrocatalytic nitrogen reduction: challenges and perspectives. Energy Environ. Sci..

[2] Choi J, Suryanto BHR, Wang D, Du HL, Hodgetts RY (2020). Identification and elimination of false positives in electrochemical nitrogen reduction studies. Nat. Commun..

[3] Shahid U, Chen Y, Gu S, Li W, Shao M (2022). Electrochemical nitrogen reduction: an intriguing but challenging quest. Trends Chem..

[4] Sun Y, Deng Z, Song XM, Li H, Huang Z (2020). Bismuth-based free-standing electrodes for ambient-condition ammonia production in neutral media. Nano-Micro Lett..

[5] Zhang W, Zhang BW (2021). Bi-atom electrocatalyst for electrochemical nitrogen reduction reactions. Nano-Micro Lett..

[6] Li X, Shen P, Luo Y, Li Y, Guo Y (2022). PdFe single-atom alloy metallene for N_2_ electroreduction. Angew. Chem. Int. Ed..

[7] Chen GF, Cao X, Wu S, Zeng X, Ding LX (2017). Ammonia electrosynthesis with high selectivity under ambient conditions via a Li^+^ incorporation strategy. J. Am. Chem. Soc..

[8] Suryanto BHR, Wang D, Azofra LM, Harb M, Cavallo L (2019). MoS_2_ polymorphic engineering enhances selectivity in the electrochemical reduction of nitrogen to ammonia. ACS Energy Lett..

[9] Chia X, Lazar P, Sofer Z, Luxa J, Pumera M (2016). Layered SnS versus SnS_2_: valence and structural implications on electrochemistry and clean energy electrocatalysis. J. Phys. Chem. C.

[10] Biswas S, Nandi N, Kamboj J, Pan AB (2021). Alteration of electronic band structure via a metal–semiconductor interfacial effect enables high faradaic efficiency for electrochemical nitrogen fixation. ACS Nano.

[11] Chu K, Luo Y, Shen P, Li X, Li Q (2022). Unveiling the synergy of O-vacancy and heterostructure over MoO_3-x_/MXene for N_2_ electroreduction to NH_3_. Adv. Energy Mater..

[12] Li L, Tang C, Xia B, Jin H, Zheng Y (2019). Two-dimensional mosaic bismuth nanosheets for highly selective ambient electrocatalytic nitrogen reduction. ACS Catal..

[13] Fu Y, Richardson P, Li K, Yu H, Yu B (2020). Transition metal aluminum boride as a new candidate for ambient-condition electrochemical ammonia synthesis. Nano-Micro Lett..

[14] Liu C, Li Q, Wu C, Zhang J, Jin Y (2019). Single-boron catalysts for nitrogen reduction reaction. J. Am. Chem. Soc..

[15] Huang Y, Yang T, Yang L, Liu R, Zhang G (2019). Graphene–boron nitride hybrid-supported single Mo atom electrocatalysts for efficient nitrogen reduction reaction. J. Mater. Chem. A Mater..

[16] Li Y, Gao D, Zhao S, Xiao Y, Guo Z (2021). Carbon doped hexagonal boron nitride nanoribbon as efficient metal-free electrochemical nitrogen reduction catalyst. Chem. Eng. J..

[17] Chen C, Yan D, Wang Y, Zhou Y, Zou Y (2019). B-N pairs enriched defective carbon nanosheets for ammonia synthesis with high efficiency. Small.

[18] Chang B, Li L, Shi D, Jiang H, Ai Z (2021). Metal-free boron carbonitride with tunable boron Lewis acid sites for enhanced nitrogen electroreduction to ammonia. Appl. Catal. B.

[19] Yu X, Han P, Wei Z, Huang L, Gu Z (2018). Boron-doped graphene for electrocatalytic N_2_ reduction. Joule.

[20] Feng Z, Tang Y, Chen W, Wei D, Ma Y (2020). O-doped graphdiyne as metal-free catalysts for nitrogen reduction reaction. Mol. Catal..

[21] Yang Y, Zhang L, Hu Z, Zheng Y, Tang C (2020). The crucial role of charge accumulation and spin polarization in activating carbon-based catalysts for electrocatalytic nitrogen reduction. Angew. Chem. Int. Ed..

[22] Shen P, Li X, Luo Y, Guo Y, Zhao X (2022). High-efficiency N_2_ electroreduction enabled by Se-vacancy-rich WSe_2–x_ in water-in-salt electrolytes. ACS Nano.

[23] Shen P, Li X, Luo Y, Zhang N, Zhao X (2022). Ultra-efficient N_2_ electroreduction achieved over a rhodium single-atom catalyst (Rh_1_/MnO_2_) in water-in-salt electrolyte. Appl. Catal. B.

[24] Zhang Q, Liu B, Yu L, Bei Y, Tang B (2020). Synergistic promotion of the electrochemical reduction of nitrogen to ammonia by phosphorus and potassium. ChemCatChem.

[25] Biswas A, Kapse S, Ghosh B, Thapa R, Dey RS (2022). Lewis acid–dominated aqueous electrolyte acting as co-catalyst and overcoming N_2_ activation issues on catalyst surface. PNAS.

[26] Nag A, Raidongia K, Hembram KPSS, Datta R, Waghmare U (2010). Graphene analogues of BN: novel synthesis and properties. ACS Nano.

[27] Biswas S, Sarkar M, Das N, Kamboj RS (2020). Dey, A no-sweat strategy for graphene-macrocycle co-assembled electrocatalyst toward oxygen reduction and ambient ammonia synthesis. Inorg. Chem..

[28] Grahame DC (1947). The electrical double layer and the theory of electrocapillarity. Chem. Rev..

[29] Zhang Y, Du H, Ma Y, Ji L, Guo H (2019). Hexagonal boron nitride nanosheet for effective ambient N_2_ fixation to NH_3_. Nano Res..

[30] Kresse G, Joubert D (1999). From ultrasoft pseudopotentials to the projector augmented-wave method. Phys. Rev. B.

[31] Blöchl PE (1994). Projector augmented-wave method. Phys. Rev. B.

[32] Perdew JP, Burke K, Ernzerhof M (1996). Generalized gradient approximation made simple. Phys. Rev. Lett..

[33] Kapse S, Janwari S, Waghmare UV, Thapa R (2021). Energy parameter and electronic descriptor for carbon based catalyst predicted using QM/ML. Appl. Catal. B.

[34] Chen X, Ong WJ, Zhao X, Zhang P, Li N (2021). Insights into electrochemical nitrogen reduction reaction mechanisms: combined effect of single transition-metal and boron atom. J. Energy Chem..

[35] Zheng J, Lyu Y, Qiao M, Veder JP, Marco RD (2019). Tuning the electron localization of gold enables the control of nitrogen-to-ammonia fixation. Angew. Chem. Int. Ed..

[36] Choutipalli VSK, Esackraj K, Subramanian V (2022). Nitrogen fixation at the edges of boron nitride nanomaterials: synergy of doping. Front. Chem..

[37] Giusto P, Arazoe H, Cruz D, Lova P, Heil T (2020). Boron carbon nitride thin films: from disordered to ordered conjugated ternary materials. J. Am. Chem. Soc..

[38] Chen S, Chen Z, Siahrostami S, Higgins D, Nordlund D (2018). Designing boron nitride islands in carbon materials for efficient electrochemical synthesis of hydrogen peroxide. J. Am. Chem. Soc..

[39] Zhou M, Wang S, Yang P, Huang C, Wang X (2018). Boron carbon nitride semiconductors decorated with CdS nanoparticles for photocatalytic reduction of CO_2_. ACS Catal..

[40] Wang X, Zhi C, Li L, Zeng H, Li C (2011). “Chemical blowing” of thin-walled bubbles: high-throughput fabrication of large-area, few-layered BN and C_x_-BN nanosheets. Adv. Mater..

[41] Huang C, Chen C, Zhang M, Lin L, Ye X (2015). Carbon-doped BN nanosheets for metal-free photoredox catalysis. Nat. Commun..

[42] Sarkar S, Biswas A, Kamboj N, Dey RS (2020). Unveiling the potential of an Fe bis(terpyridine) complex for precise development of an Fe-N-C electrocatalyst to promote the oxygen reduction reaction. Inorg. Chem..

[43] Chen L, Zhou M, Luo Z, Wakeel M, Asiri AM (2019). Template-free synthesis of carbon-doped boron nitride nanosheets for enhanced photocatalytic hydrogen evolution. Appl. Catal. B.

[44] Florent M, Bandosz TJ (2018). Irreversible water mediated transformation of BCN from a 3D highly porous form to its nonporous hydrolyzed counterpart. J. Mater. Chem. A Mater..

[45] Tang C, Bando Y, Huang Y, Zhi C, Golberg D (2008). Synthetic routes and formation mechanisms of spherical boron nitride nanoparticles. Adv. Funct. Mater..

[46] Beniwal S, Hooper J, Miller DP, Costa PS, Chen G (2017). Graphene-like boron–carbon–nitrogen monolayers. ACS Nano.

[47] Sarkar S, Biswas A, Siddharthan EE, Thapa R, Dey RS (2022). Strategic modulation of target-specific isolated Fe, Co single-atom active sites for oxygen electrocatalysis impacting high power Zn–air battery. ACS Nano.

[48] Matsoso J, Ranganathan K, Mutuma BK, Lerotholi T, Jones G (2017). Synthesis and characterization of boron carbon oxynitride films with tunable composition using methane, boric acid and ammonia. New J. Chem..

[49] Gu D, Zhou Y, Ma R, Wang F, Liu Q (2018). Facile synthesis of N-doped graphene-like carbon nanoflakes as efficient and stable electrocatalysts for the oxygen reduction reaction. Nano-Micro Lett..

[50] Terminello LJ, Chaiken A, Lapiano-Smith DA, Doll GL, Sato T (1998). Morphology and bonding measured from boron-nitride powders and films using near-edge x-ray absorption fine structure. J. Vacuum Sci. Technol. A.

[51] Li Q, Shen P, Tian Y, Li X, Chu K (2022). Metal-free BN quantum dots/graphitic C_3_N_4_ heterostructure for nitrogen reduction reaction. J. Colloid Interface Sci..

[52] Hemraj-Benny T, Banerjee S, Sambasivan S, Fischer DA, Han W (2005). Investigating the structure of boron nitride nanotubes by near-edge X-ray absorption fine structure (NEXAFS) spectroscopy. Phys. Chem. Chem. Phys..

[53] Frati F, Hunault MOJY, Groot FMF (2020). Oxygen K-edge X-ray absorption spectra. Chem. Rev..

[54] Watanabe MO, Itoh S, Mizushima K, Sasaki T (1998). Bonding characterization of BC_2_N thin films. Appl. Phys. Lett..

[55] Terauchi M, Tanaka M, Matsumoto T, Saito Y (1998). Electron energy-loss spectroscopy study of the electronic structure of boron nitride nanotubes. J. Electron. Microsc..

[56] Brühwiler PA, Maxwell AJ, Puglia C, Nilsson A, Andersson S (1995). π* and σ* excitons in C 1s absorption of graphite. Phys. Rev. Lett..

[57] Lazouski N, Steinberg KJ, Gala ML, Krishnamurthy D, Viswanathan V (2022). Proton donors induce a differential transport effect for selectivity toward ammonia in lithium-mediated nitrogen reduction. ACS Catal..

[58] Krempl K, Pedersen JB, Kibsgaard J, Vesborg PCK, Chorkendorff I (2022). Electrolyte acidification from anode reactions during lithium mediated ammonia synthesis. Electrochem. Commun..

[59] Guo Y, Gu J, Zhang R, Zhang S, Li Z (2021). Molecular crowding effect in aqueous electrolytes to suppress hydrogen reduction reaction and enhance electrochemical nitrogen reduction. Adv. Energy Mater..

[60] Liu Z, Zhang M, Wang H, Cang D, Ji X (2020). Defective carbon-doped boron nitride nanosheets for highly efficient electrocatalytic conversion of N_2_ to NH_3_. ACS Sustain. Chem. Eng..

[61] Chu K, Li X, Tian Y, Li Q, Guo Y (2021). Boron nitride quantum dots/Ti_3_C_2_T_x_-MXene heterostructure for efficient electrocatalytic nitrogen fixation. Energy Environ. Mater..

[62] Zhang Q, Luo F, Ling Y, Xiao S, Li M (2020). Identification of functionality of heteroatoms in boron, nitrogen and fluorine ternary-doped carbon as a robust electrocatalyst for nitrogen reduction reaction powered by rechargeable zinc–air batteries. J. Mater. Chem. A Mater..

[63] Yesudoss K, Lee G, Shanmugam S (2021). Strong catalyst support interactions in defect-rich γ-Mo_2_N nanoparticles loaded 2D-h-BN hybrid for highly selective nitrogen reduction reaction. Appl. Catal. B.

[64] Chu K, Li X, Li Q, Guo Y, Zhang H (2021). Synergistic enhancement of electrocatalytic nitrogen reduction over boron nitride quantum dots decorated Nb_2_CT_x_-MXene. Small.

[65] Shi L, Yin Y, Wang S, Sun H (2020). Rational catalyst design for N_2_ reduction under ambient conditions: strategies toward enhanced conversion efficiency. ACS Catal..

[66] He C, Wu ZY, Zhao L, Ming M, Zhang Y (2019). Identification of FeN_4_ as an efficient active site for electrochemical N_2_ reduction. ACS Catal..

[67] Ghorai UK, Paul S, Ghorai B, Adalder A, Kapse S (2021). Scalable production of cobalt phthalocyanine nanotubes: efficient and robust hollow electrocatalyst for ammonia synthesis at room temperature. ACS Nano.

[68] Murmu S, Paul S, Kapse S, Thapa R, Chattopadhyay S (2021). Unveiling the genesis of the high catalytic activity in nickel phthalocyanine for electrochemical ammonia synthesis. J. Mater. Chem. A.

