# Supplementary material for: Oxygen Functionalization-Induced Charging Effect on Boron Active Sites for High-Yield Electrocatalytic NH3 Production
Source: Nanomicro Lett. 2022 Nov 5;14:214. doi: 10.1007/s40820-022-00966-7 (PMC9637079; doi:10.1007/s40820-022-00966-7)
Supplement: Supplementary file 1 — Supplementary file1 (PDF 2350 KB) [file 40820_2022_966_MOESM1_ESM.pdf]

Supporting Information for

Oxygen Functionalization Induced Charging Effect on Boron Active Sites for High-Yield Electrocatalytic NH_3 Production

Ashmita Biswas[1], Samadhan Kapse[2], Ranjit Thapa[2], Ramendra Sundar Dey[1, *]

[1]Institute of Nano Science and Technology (INST), Sector-81, Mohali-140306, Punjab, India

[2]Department of Physics, SRM University – AP, Amaravati 522240, Andhra Pradesh, India

*Corresponding author. E-mail: rsdey@inst.ac.in (Ramendra Sundar Dey)

S1 Experimental Section

S1.1 Materials

Melamine, boric acid, urea, ammonium chloride (NH_4Cl), hydrazine monohydrate ($N_2H_4·H_2O$), para- (dimethylamino)benzaldehyde, potassium hydroxide (KOH), sodium hydroxide (NaOH), trisodium citrate, sodium hypochlorite (NaClO), sodium nitroferricyanide ($Na_2[Fe(NO)(CN)_5]$) and maleic acid were all purchased from Sigma Aldrich. Hydrochloric acid (HCl), sulfuric acid (H_2SO_4), phosphoric acid (H_3PO_4), ethanol and DMSO-D^6 were purchased from Merck chemicals India. All the chemicals used were at least of analytical grade and were used without any further purification. All aqueous solution was prepared using Millipore water. $^{14}N_2$ cylinder was purchased from Sigma gases and $^{15}N_2$ gas cylinder was purchased from Sigma Aldrich.

S1.2 Ex-*situ* Material Characterizations

The surface morphological characterization along with the determination of the elemental composition of $BNCO_{(1000)}$ catalyst sample was investigated using field emission scanning electron microscopy (FESEM) and high-resolution transmission electron microscopy (HRTEM) analysis, which were carried out on JEOL JSM-7600F and JEM2100 instruments along with selected area electron diffraction (SAED). X-ray diffraction (XRD) study was carried out on a Bruker D8 advances instrument using Cu-Kα (λ = 1.5406 Å) radiation in the 2θ range from 10° to 80° with an acceleration voltage of 40 kV. The chemicals bonds present in the catalysts were evaluated with Fourier transform infra-red spectroscopy (FTIR), carried out on FTIR BRUKER VERTEX 70 Sr. 2236 FTIR instrument at room temperature. The surface elemental composition and bonding configuration of the prepared samples was determined using X-ray photoelectron spectroscopy (XPS) spectrometer (K-Alpha 1063) instruments in an ultrahigh vacuum chamber ($7X10^{-9}$ torr) using Al-K_α radiation (1486.6 eV). The NEXFAS spectra were measured in Beamline at Indus-2 (DMP/MG), UGC-DAE CSR, Indore under an ultra-high vacuum (UHV) chamber maintained at base pressure of $5x10^{-10}$ mbar. The measurements were taken in the total electron yield (TEY) mode. UV-vis characterizations were performed in UV−vis−NIR (Cary 5000 UV−vis−NIR, Model: G9825A CARY) spectrophotometer that has the ability to measure 300−2800 nm by a Pb Smart NIR detector. ^{1}H-nuclear magnetic resonance (NMR) spectra was measured with a 400 MHz Bruker Avance II 400 NMR spectrometer. Chemical shifts are reported in parts per million (δ) calibrated by using tetramethylsilane as an internal standard for samples in [D^6]DMSO. All the pH measurements were done using Eutech pH meter instrument. All the electrochemical measurements were performed in CHI 760E and BioLogic VSP potentiostats.

S1.3 Electrode Modification

10 mg of the $BNCO_{(1000)}$ catalyst was dispersed in a 1:1 (v/v) mixture of water and isopropyl alcohol (1 mL) and ultrasonicated for 30 min. This same ink solution was used for all the electrochemical studies involved in NRR. Before modifying the glassy-carbon electrode (GCE) with the catalyst ink, it was properly cleaned with 1, 0.3 and 0.05 µm alumina (Al_2O_3) powder and ultrasonicated with deionised water for 3 min. Furthermore, cyclic voltammetry (CV) was

performed for 100 cycles in 0.5 M H_2SO_4 at 100 mV s^{-1} scan rate in order to remove all form of impurities from the catalyst surface. The glassy carbon part of the electrodes was then dropcasted with 0.5 mg cm^{-2} of the catalyst ink and dried overnight under vacuum. This same mass loading was maintained for all the control samples investigated in this work.

S1.4 $^{15}N_2$- isotope Labeling Experiments

$^{15}N_2$ (Sigma Aldrich, 98 atom% $^{15}N_2$) was used as the feeding gas in the labelling experiment. Before feeding the electrolyte solution with $^{15}N_2$, the electrolyte was degassed using Ar for an hour. A low-velocity gas flow system was adopted due to the limited supply and expense of $^{15}N_2$. After electrolysis at -0.1 V vs. RHE for 5 h, the electrolyte was taken out and concentrated, followed by addition of 0.01 M maleic acid and 0.4 ml of D^6-DMSO. The produced ammonia was quantified using ^1H nuclear magnetic resonance measurements in a 400 MHz Bruker Advance II 400 NMR spectrometer.

S2 Supplementary Figures and Tables

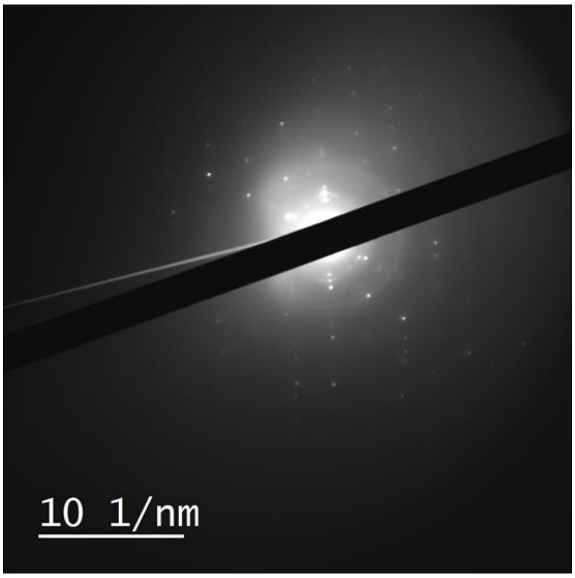

Fig. S1 SAED pattern of $BNCO_{(1000)}$ catalyst

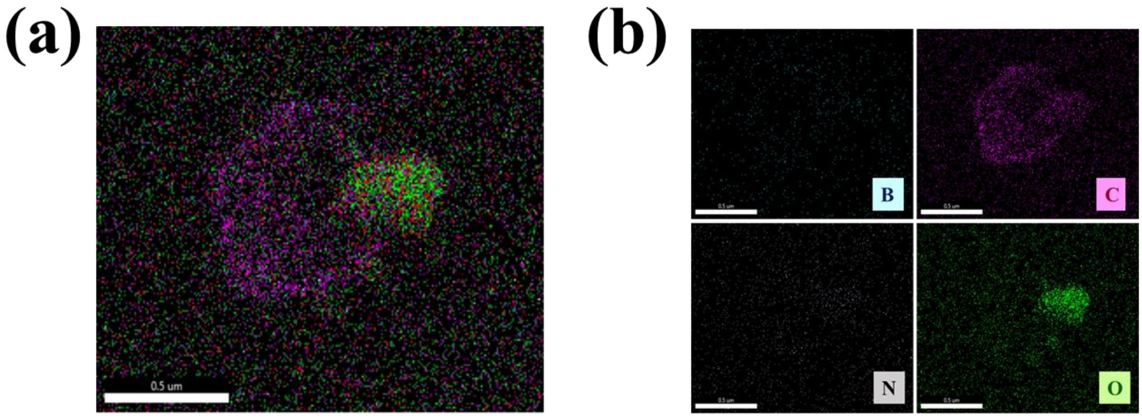

Fig. S2 Elemental mapping of $BNCO_{(1000)}$ catalyst showing (**a**) all elements combined and (**b**) distinctly B, C, N and O

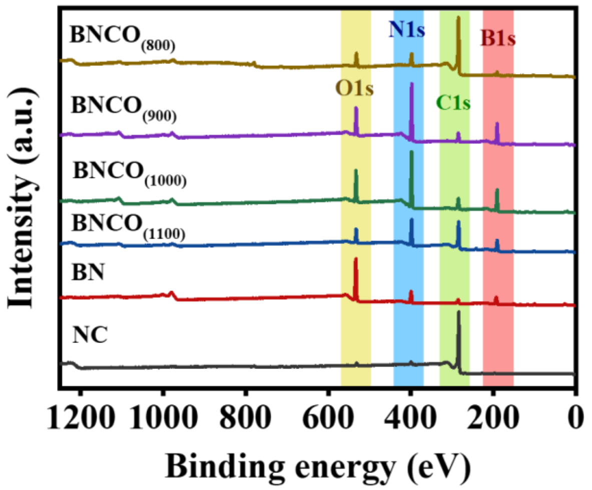

Fig. S3 XPS full survey spectra of all the synthesized catalysts showing B 1s, C 1s, O 1s and N 1s peaks

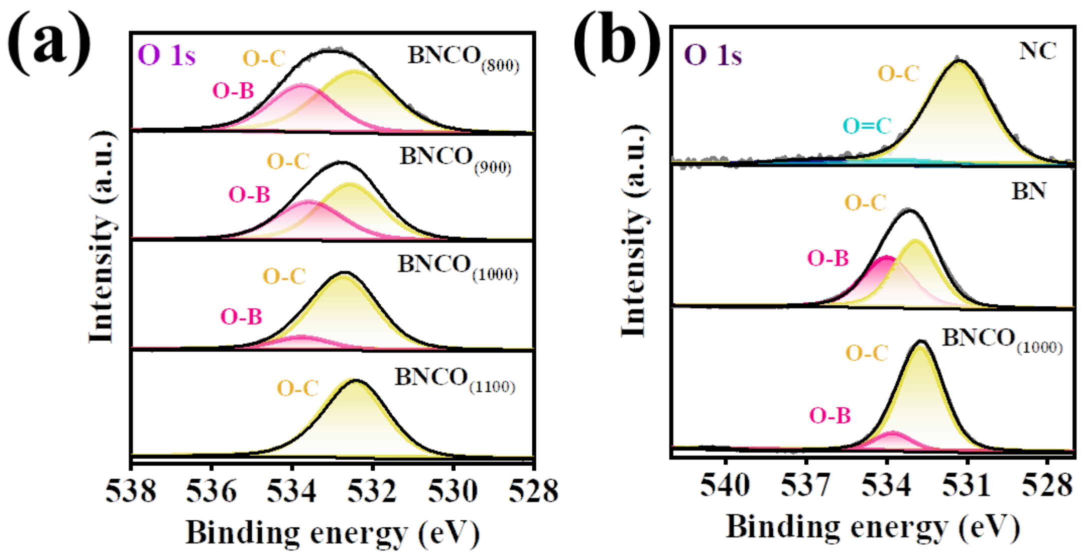

Fig. S4 Comparative deconvoluted O 1s spectra of (**a**) BNCO catalyst synthesized at different pyrolysis temperature, (**b**) control samples like NC, BN and final $BNCO_{(1000)}$ catalyst

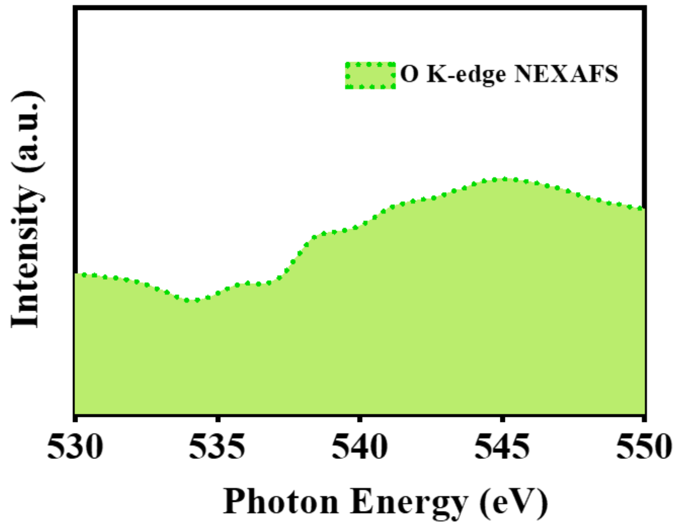

Fig. S5 O K-edge NEXAFS spectrum of $BNCO_{(1000)}$ catalyst

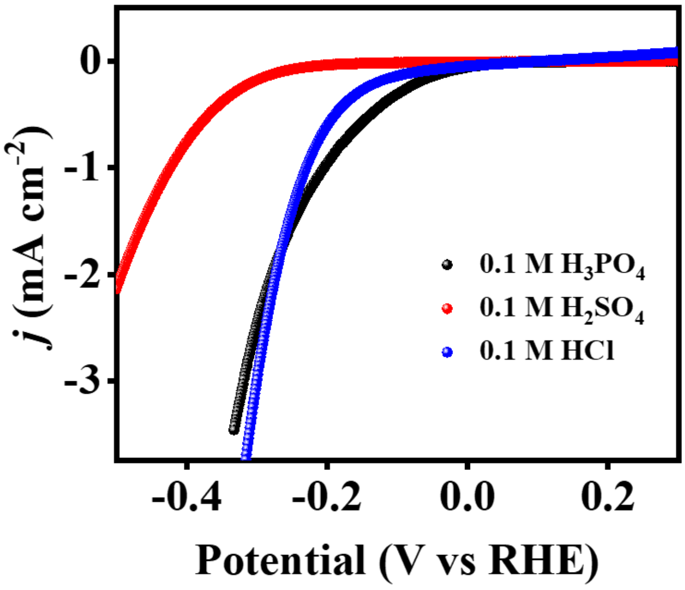

Fig. S6 Comparative LSV curves of $BNCO_{(1000)}$ catalyst in N_2 purged electrolytes at 10 mV s^{-1} scan rate

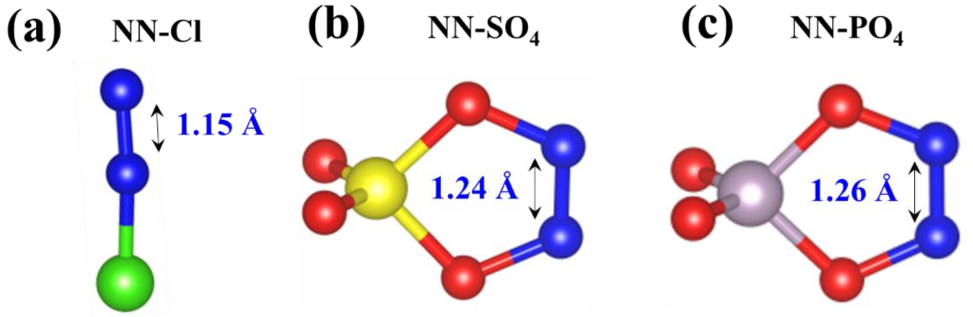

Fig. S7 N≡N bond length of the N_2 molecule bonding with (**a**) Cl (**b**) SO_4 (**c**) PO_4 anions. The blue, red, green, yellow, pink colored spheres represent the nitrogen, oxygen, chlorine, Sulphur, phosphorous atoms respectively

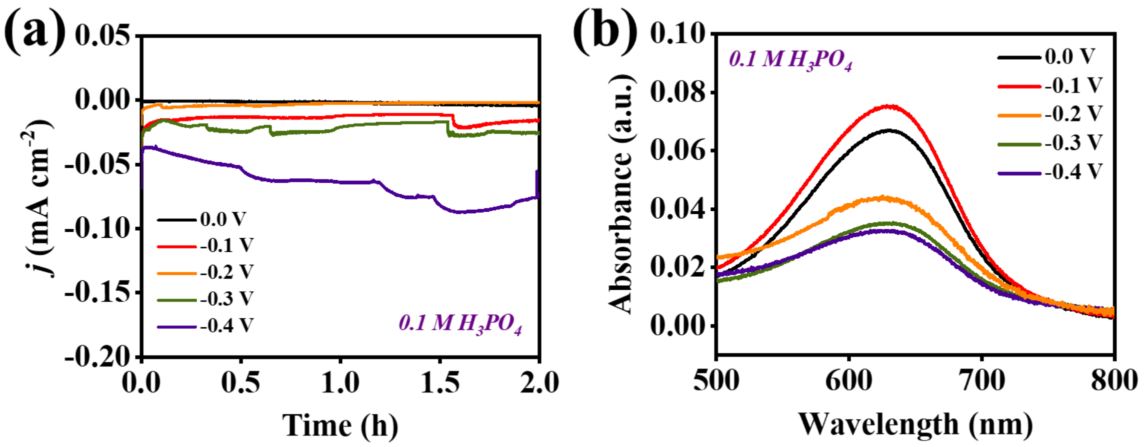

Fig. S8 (**a**) Potential dependent chronoamperometric response of $BNCO_{(1000)}$ catalyst for 2 h run (each) in 0.1 M H_3PO_4 electrolyte. (**b**) UV−vis absorption spectra of the electrolyte (0.1 M H_3PO_4 with dissolved NH_4^+) stained with indophenol blue indicator at different potentials after 2 h of incubation from the chronoamperometric run

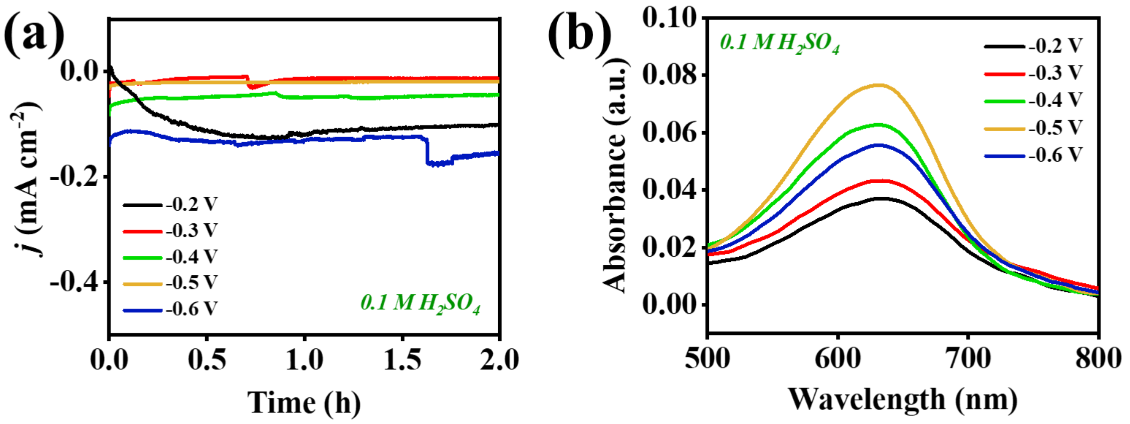

Fig. S9 (**a**) Potential dependent chronoamperometric response of $BNCO_{(1000)}$ catalyst for 2 h run (each) in 0.1 M H_2SO_4 electrolyte. (**b**) UV−vis absorption spectra of the electrolyte (0.1 M H_2SO_4 with dissolved NH_4^+) stained with indophenol blue indicator at different potentials after 2 h of incubation from the chronoamperometric run

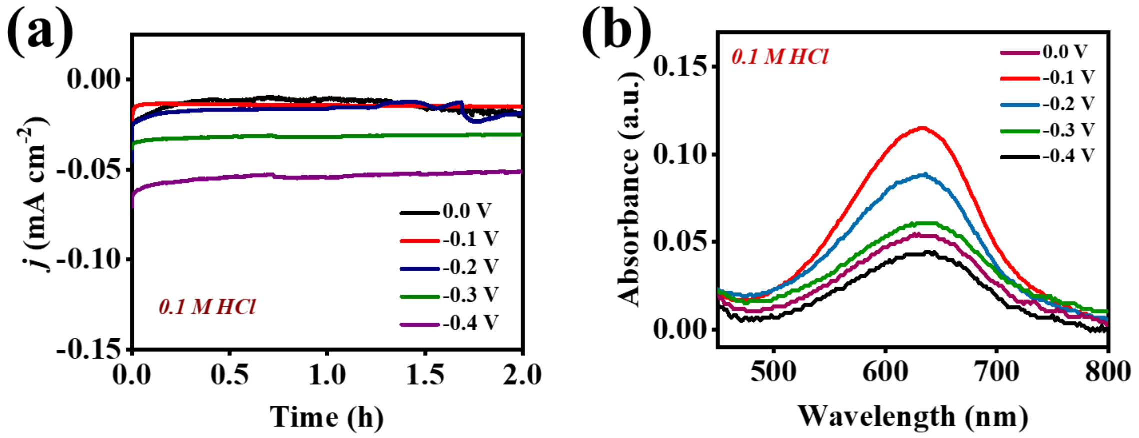

Fig. S10 (**a**) Potential dependent chronoamperometric response of $BNCO_{(1000)}$ catalyst for 2 h run (each) in 0.1 M HCl electrolyte. (**b**) UV−vis absorption spectra of the electrolyte (0.1 M HCl with dissolved NH_4^+) stained with indophenol blue indicator at different potentials after 2 h of incubation from the chronoamperometric run

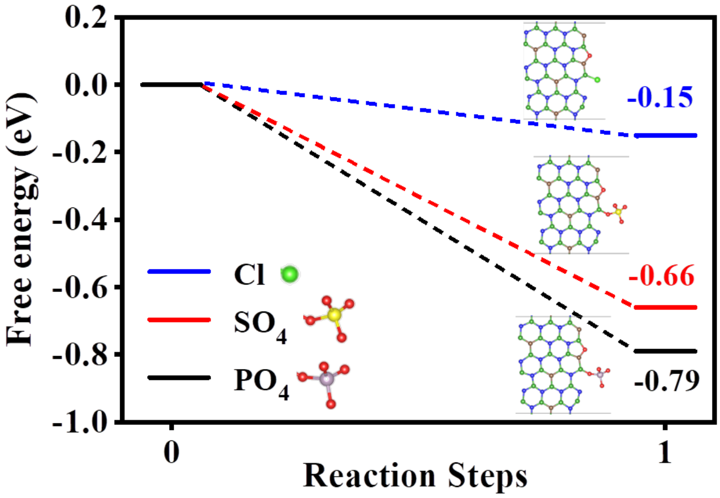

Fig. S11 Free energy diagram of adsorption of Cl, SO_4 and PO_4 on edge B site of $BNCO_{(1000)}$ system; inset shows the optimised model structures of (**a**) Cl (**b**) SO_4 (**c**) PO_4 adsorbed BNCO models

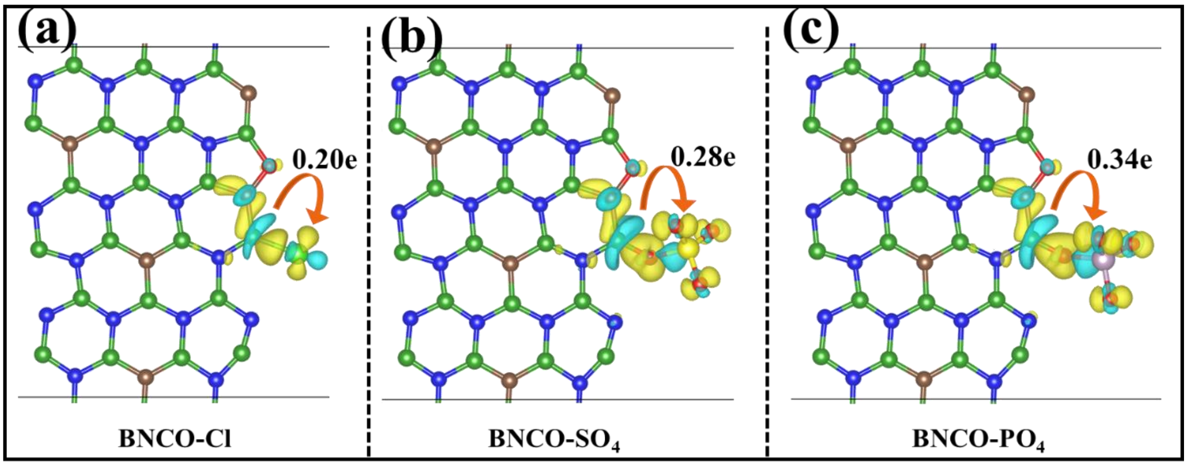

Fig. S12 Charge density difference analysis of (**a**) Cl-adsorbed, (**b**) SO_4-adsorbed and (**c**) PO_4-adsorbed BNCO system. Yellow and blue lobes indicate electron accumulation and depletion, respectively (Isosurface value = 0.001 e Å^{-3})

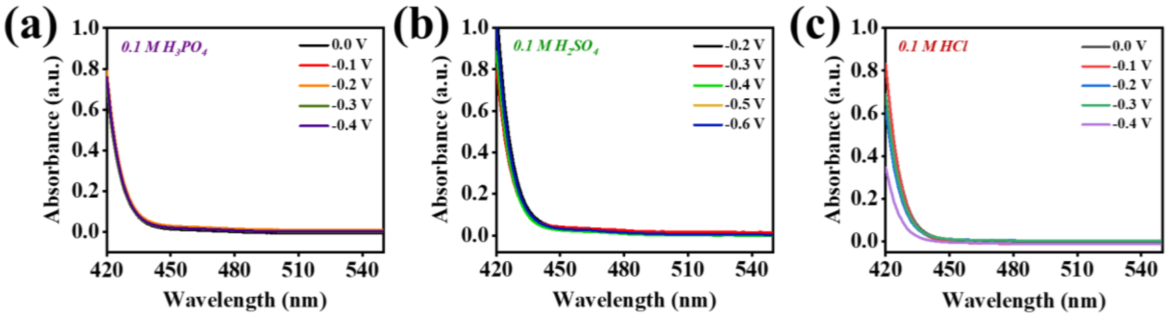

Fig. S13 UV-vis absorption spectra for the detection of N_2H_4 at different applied potentials for 2h of NRR measurements in (**a**) H_3PO_4, (**b**) H_2SO_4 and (**c**) HCl electrolytes (0.1 M each)

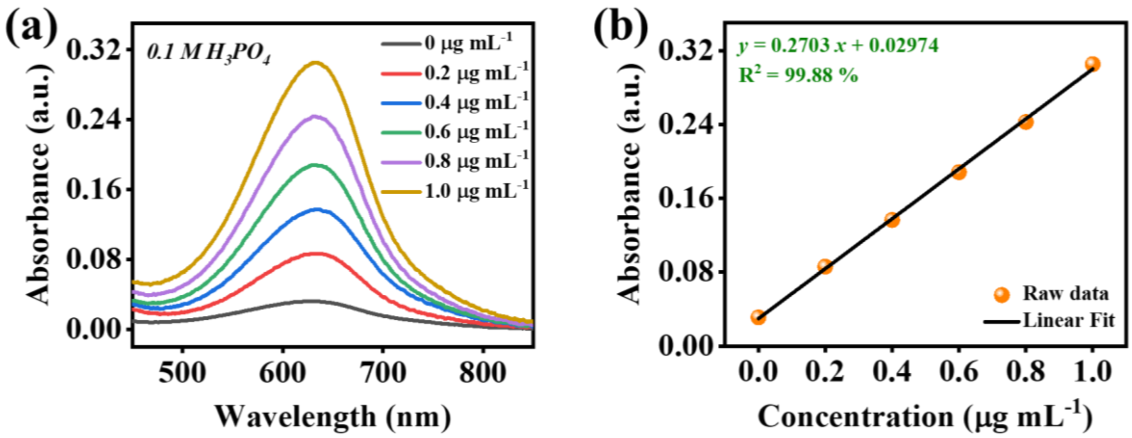

Fig. S14 (**a**) UV-vis spectra of 0.1 M H_3PO_4 solutions, representing different known concentrations of NH_4^+ stained with indophenol blue indicator solutions after 2 h incubation at room temperature and (**b**) corresponding absorbance calibration plot used in this study

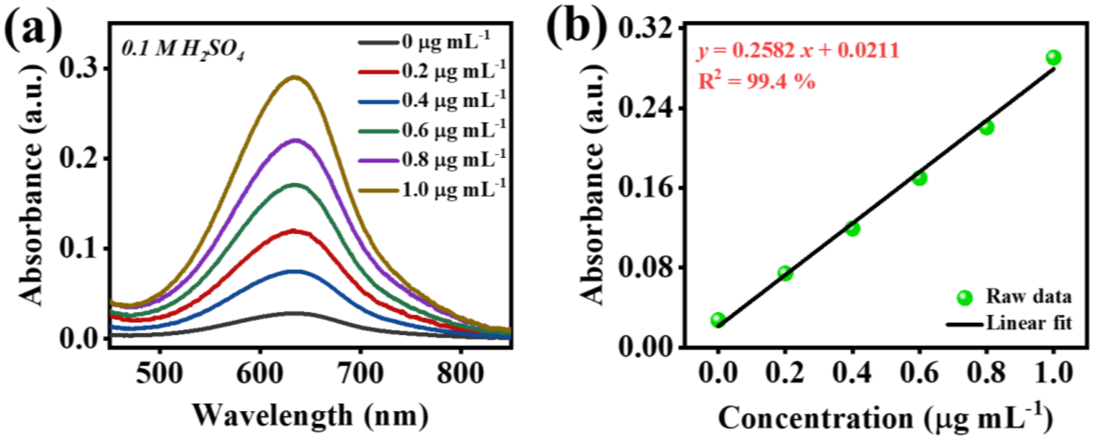

Fig. S15 (**a**) UV-vis spectra of 0.1 M H_2SO_4 solutions, representing different known concentrations of NH_4^+ stained with indophenol blue indicator solutions after 2 h incubation at room temperature and (**b**) corresponding absorbance calibration plot used in this study

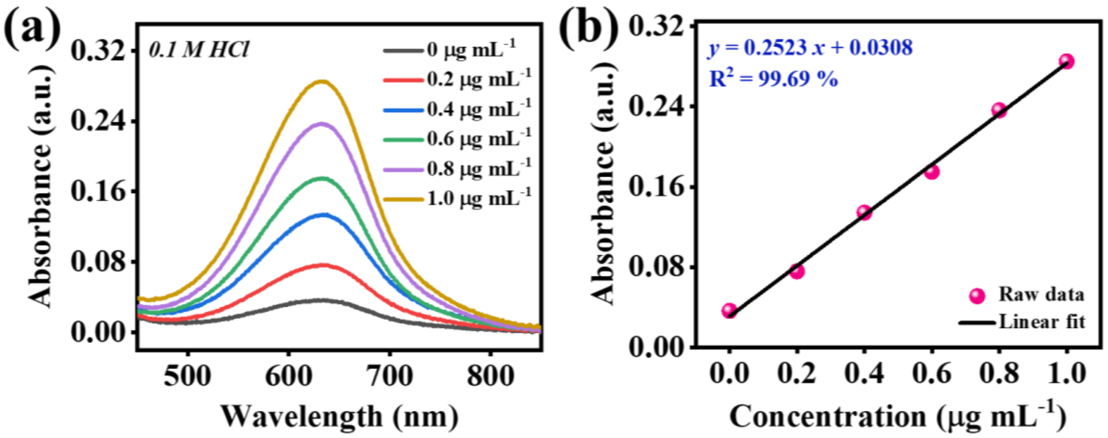

Fig. S16 (**a**) UV-vis spectra of 0.1 M HCl solutions, representing different known concentrations of NH_4^+ stained with indophenol blue indicator solutions after 2 h incubation at room temperature and (**b**) corresponding absorbance calibration plot used in this study

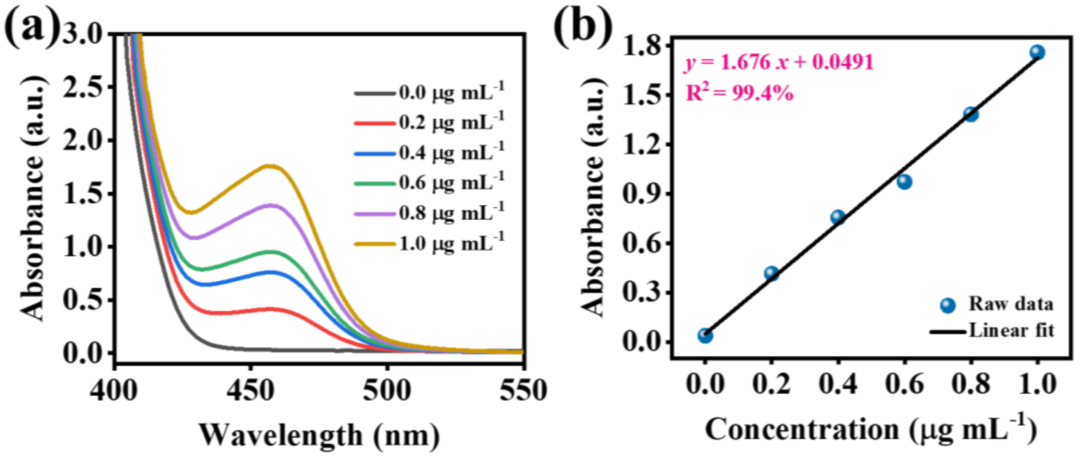

Fig. S17 (**a**) UV-vis spectra at 460 nm, representing different known concentrations of N_2H_4 after 15 min incubation at room temperature in 0.1 M HCl. (**b**) corresponding absorbance calibration plot used in this study, inset represents the colour of the solutions having different N_2H_4 concentrations, after 15 min of incubation

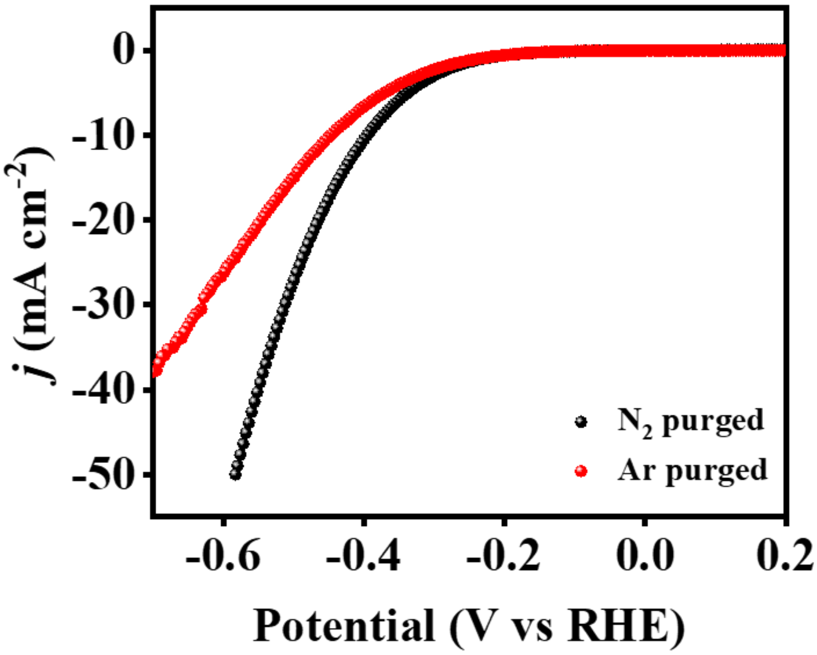

Fig. S18 Comparative LSV curves of $BNCO_{(1000)}$ catalyst in Ar and N_2 purged 0.1 M HCl at 10 mV s^{-1} scan rate

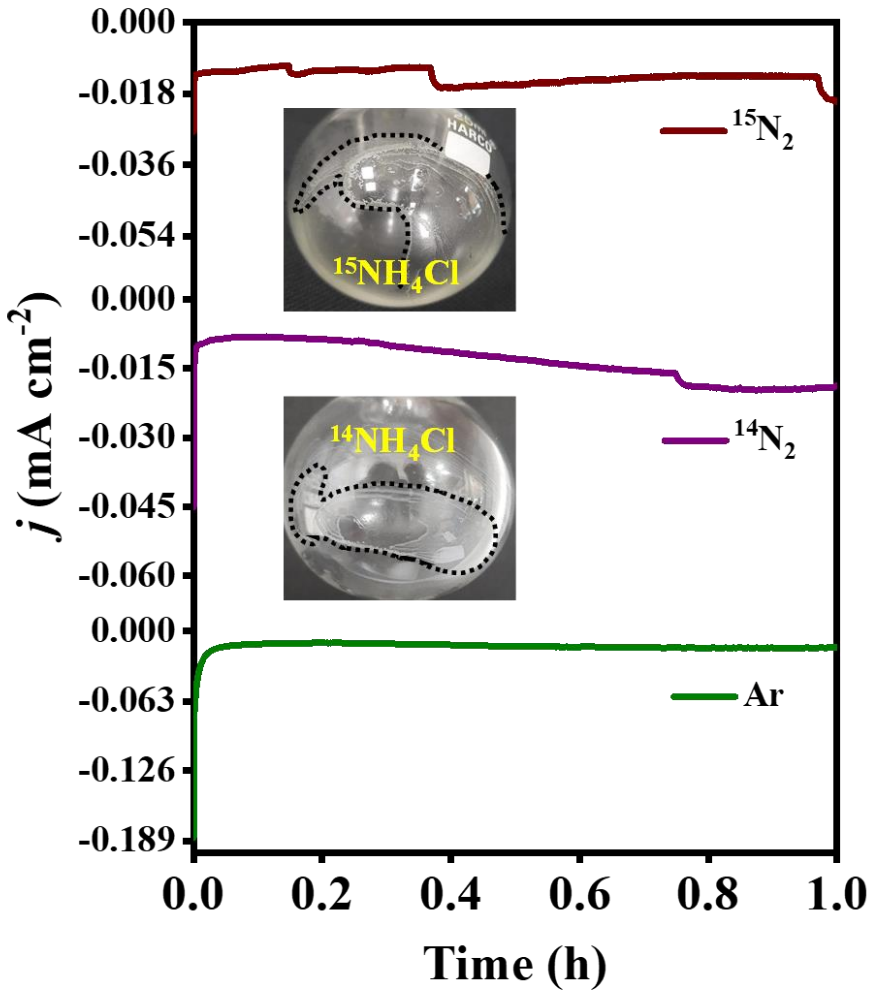

Fig. S19 Chronoamperometric response of $BNCO_{(1000)}$ catalyst for 1 h at -0.1 V vs RHE in $^{15}N_2$, $^{14}N_2$ and Ar saturated 0.1 M HCl

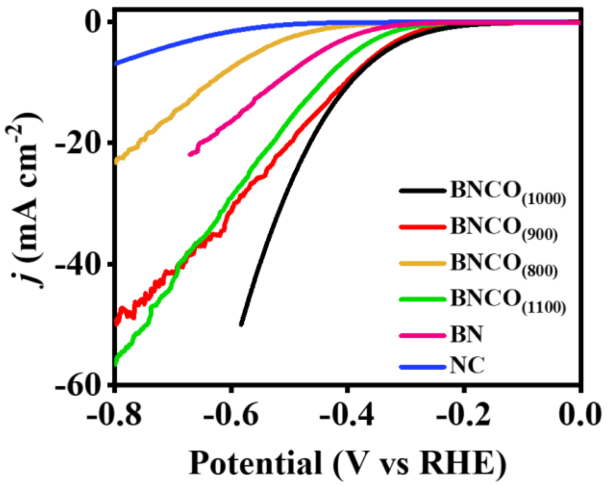

Fig. S20 LSV polarization curves of all the synthesized catalysts in N_2 fed 0.1 M HCl solution at 10 mV s^{-1} scan rate

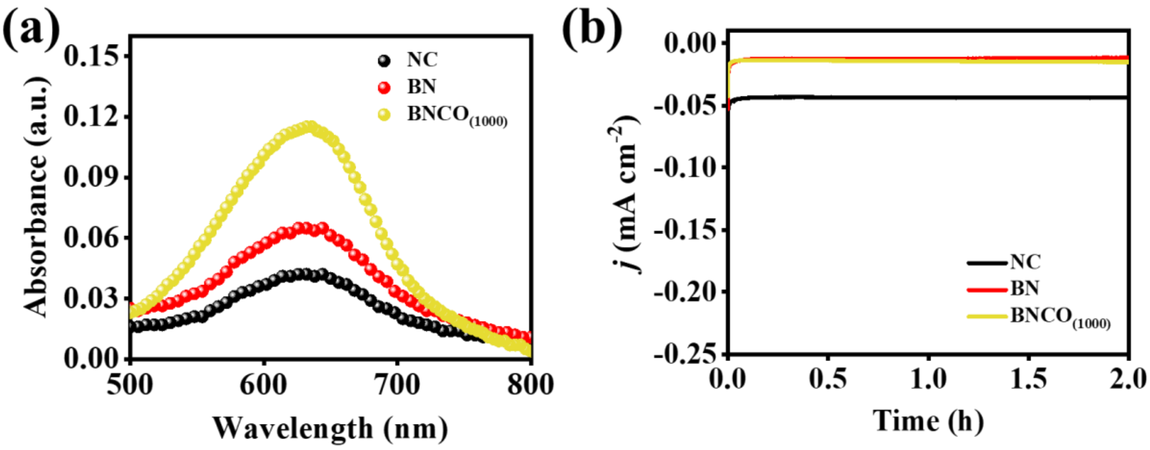

Fig. S21 (**a**) UV-visible spectra and (**b**) chronoamperometric measurements of different control samples like pristine NC and BN and comparison of NRR performance with the final $BNCO_{(1000)}$ catalyst

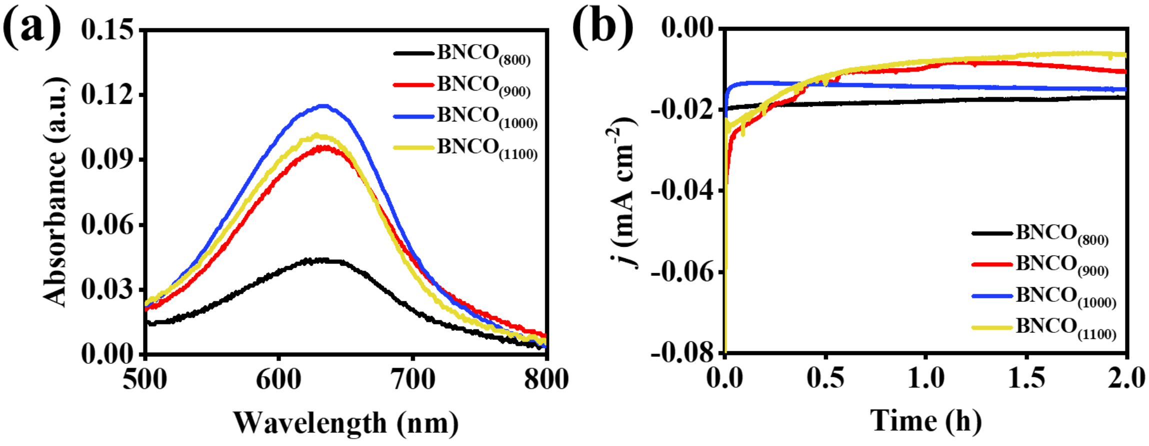

Fig. S22 (**a**) UV-visible spectra and (**b**) chronoamperometric measurements of different BNCO samples synthesized at different pyrolysis temperatures

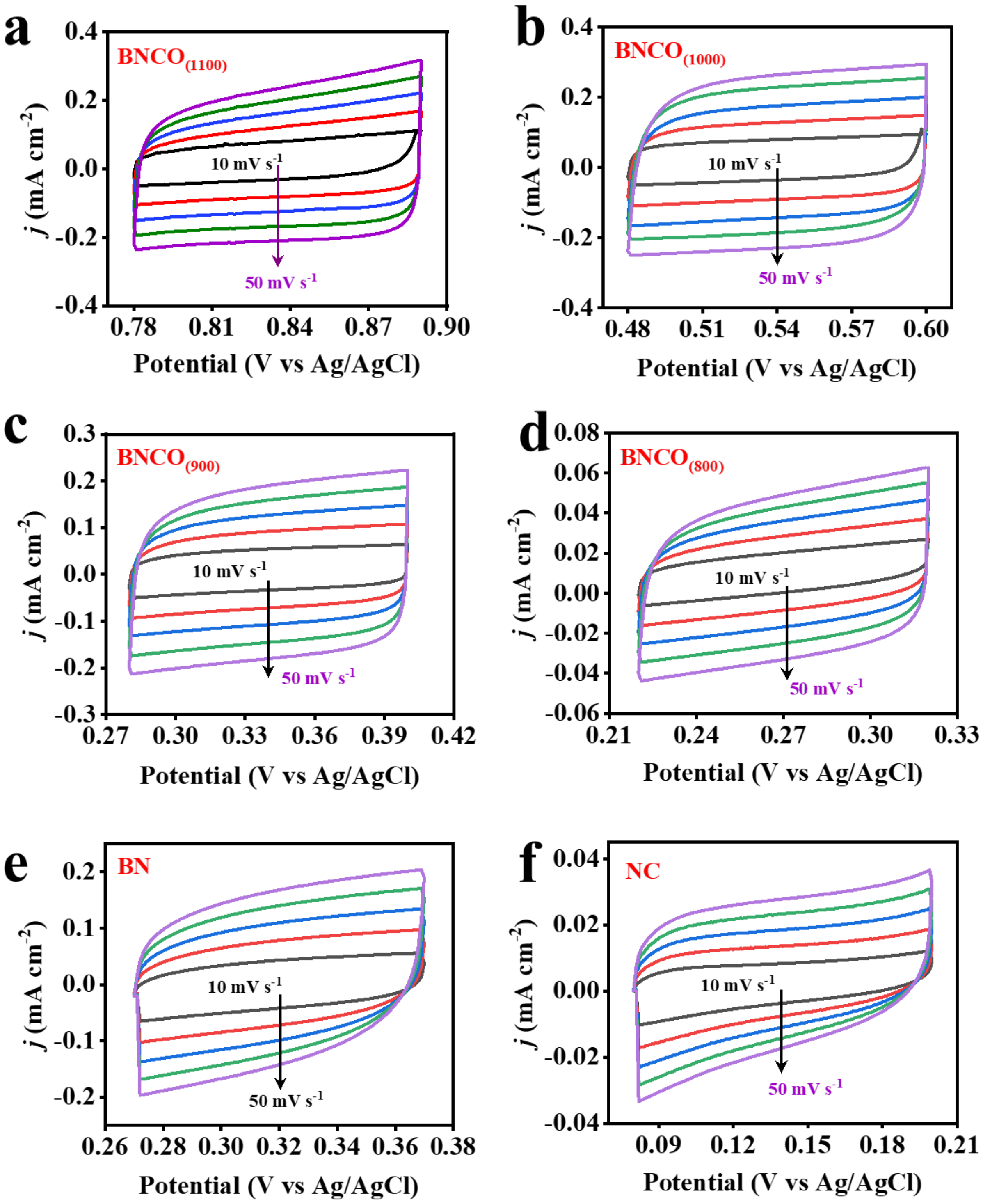

Fig. S23 (**a-f**) CV curves in the non-Faradaic region at various scan rates (10 to 50 mV s $^{-1}$) for all the synthesized catalysts and (**f**) Linear fits of difference of anodic and cathodic current densities extracted from the cyclic voltammetry curves for all the synthesized catalysts with respect to different scan rates at definite potentials, where the slope represents twice the double layer capacitance (C_{dl})

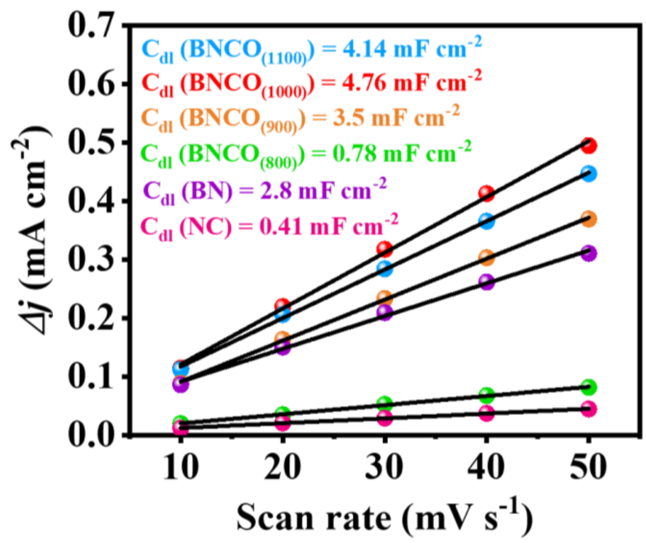

Fig. S24 Linear fits of difference of anodic and cathodic current densities extracted from the cyclic voltammetry curves for all the synthesized catalysts with respect to different scan rates at definite potentials, where the slopes represent twice the double layer capacitance (C_{dl})

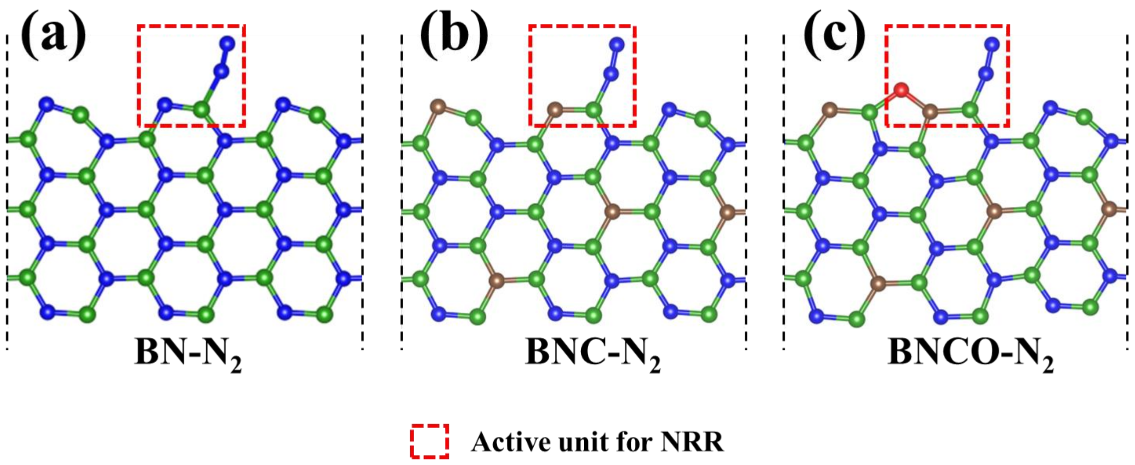

Fig. S25 Optimised model structures of N_2 adsorbed (**a**) BN (**b**) BNC (**c**) BNCO systems. The square of dotted line with red colour represents the active configuration in respective models. The N, C, O, B atoms denoted with blue, wine, red, green colour spheres respectively

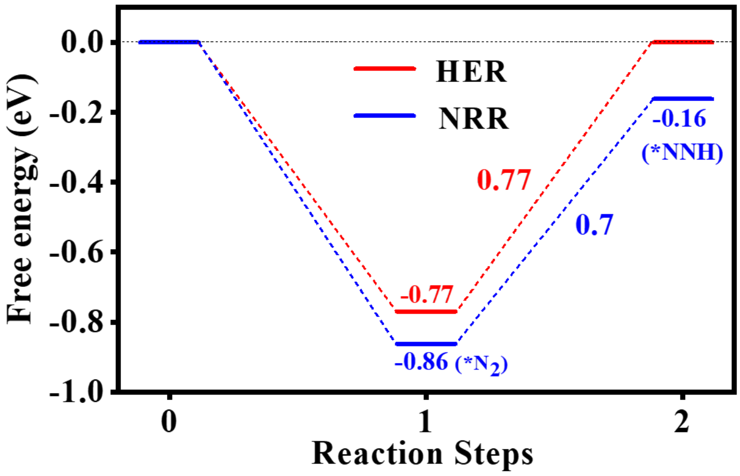

Fig. S26 Comparison of HER and NRR free energy profile for boron active site of BNCO catalyst

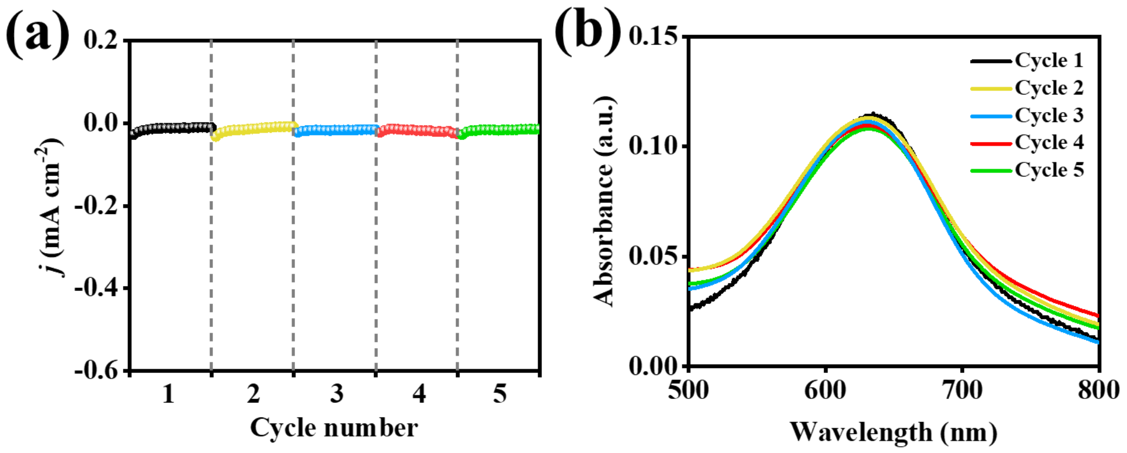

Fig. S27 (**a**) Chronoamperometric response of $BNCO_{(1000)}$ in N_2 saturated 0.1 M HCl at -0.1 V vs RHE for five consecutive cycles (each for 2h). (**b**) UV-vis absorption spectra of the electrolyte for detection of the evolved NH_3 for five consecutive chronoamperometric cycles (each for 2h) at -0.1 V vs RHE

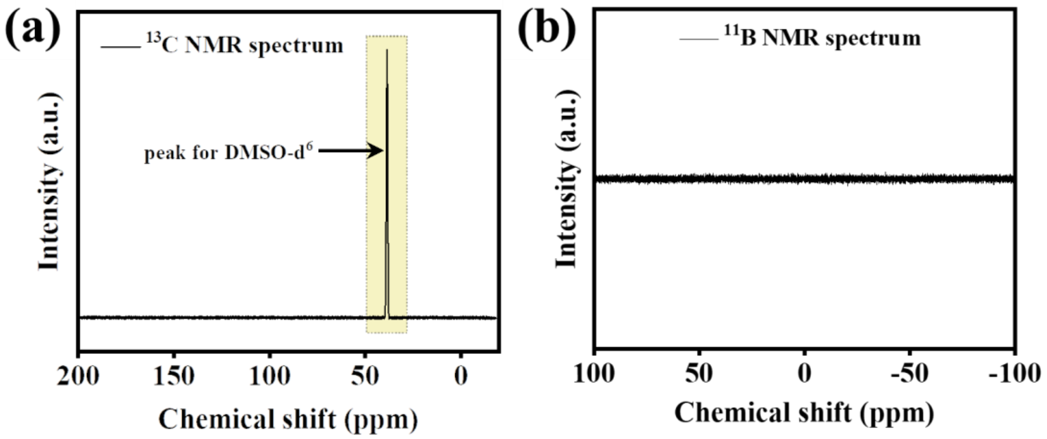

Fig. S28 Post-cycling NMR studies of the 0.1 M HCl electrolyte showing (**a**) ^{13}C and (**b**) ^{11}B NMR spectra in DMSO-d^6 solvent in a 400 MHz NMR spectrophotometer

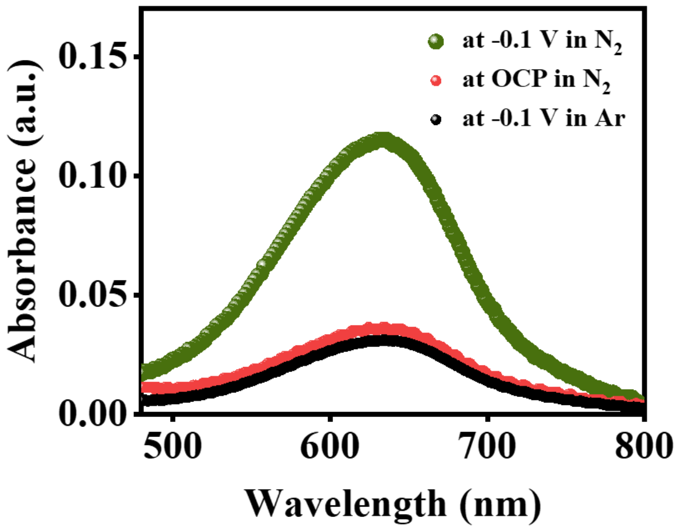

Fig. S29 UV-vis spectroscopic data of the controlled samples to verify the source and authentication of ammonia formation

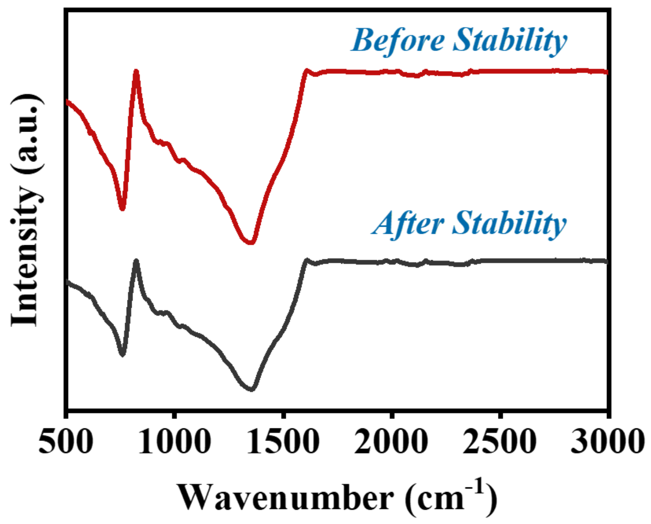

Fig. S30 FTIR spectra of $BNCO_{(1000)}$ catalyst before and after stability measurements for 48 h

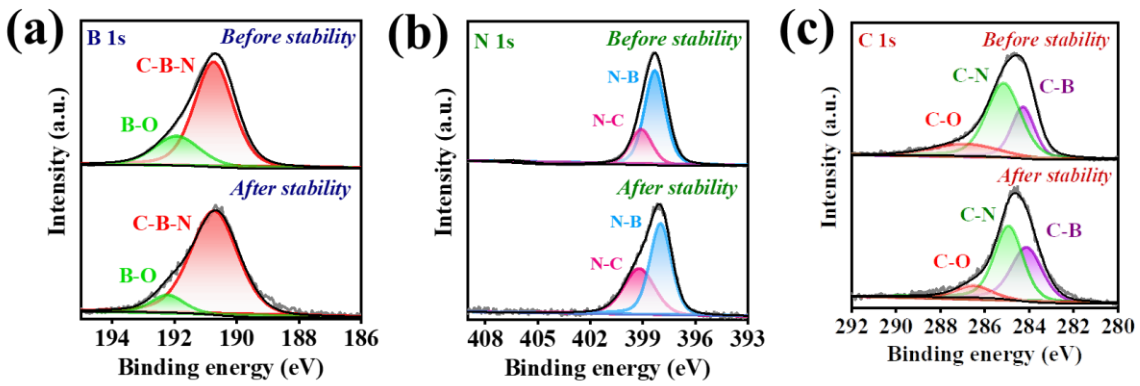

Fig. S31 Before and after stability deconvoluted XPS narrow spectra of $BNCO_{(1000)}$ catalyst at (**a**) B 1s, (**b**) N 1s and (**c**) C 1s

Table S1 Atomic % of all the elements comprising the BNCO catalyst and all the control samples from XPS analysis

	**Boron (B 1s)**	**Nitrogen (N 1s)**	**Carbon (C 1s)**	**Oxygen (O 1s)**
**NC**	-	5.2	92.46	2.34
**BN**	42.49	29.28	-	28.23
**$BNCO_{(800)}$**	9.84	10.66	71.38	8.12
**$BNCO_{(900)}$**	45.86	31.74	10.42	11.98
**$BNCO_{(1000)}$**	47.71	28.18	15.48	8.6
**$BNCO_{(1100)}$**	36.13	19.57	36.9	7.4

Table S2 Electrocatalytic NRR performance of $BNCO_{(1000)}$ catalyst in different electrolyte conditions at varying potentials

**Electrolyte**	**Potential (V vs. RHE)**	**NH_3 Yield ($\mu g\ h^{-1}\ mg_{cat}^{-1}$)**	**FE (%)**	**Mass-normalized Production rate (mmol $h^{-1}\ g_{cat}^{-1}$)**
0.1 M HCl	0.0	56.314	9.57	3.31
	**-0.1**	**211.5**	**34.72**	**12.44**
	-0.2	142.71	20.03	8.39
	-0.3	76.5	5.81	4.5
	-0.4	31.30	1.37	1.84
0.1 M H_2SO_4	-0.2	39.58	0.91	2.32
	-0.3	55.73	7.85	3.27
	-0.4	103.05	16.55	6.06

	-0.5	137.37	32.74	8.08
	-0.6	86.14	15.16	5.06
0.1 M H_3PO_4	0.0	88.07	14.7	5.18
	-0.1	107.35	13.81	6.31
	-0.2	33.42	5.23	1.96
	-0.3	13.17	1.41	0.77
	-0.4	6.49	0.24	0.38

Table S3 Comparison of NRR performance of the reported BNC class of electrocatalyst

**Catalysts**	**Electrolyte**	**Potential (V vs RHE)**	**NH_3 yield (μg h^{-1} mg_{cat}^{-1})**	**Refs.**
Mo_2N-BN	0.1 M Na_2SO_4	-0.3	37	[S1]
BCN	0.1 M HCl	-0.3	7.75	[S2]
BNQDs/$Ti_3C_2T_x$		-0.4	52.8	[S3]
C-BN	0.1 M HCl	-0.55	36.7	[S4]
h-BNNs	0.1 M HCl	-0.75	22.4	[S5]
(BNQDs)/graphitic C_3N_4	0.5 M $LiClO_4$	-0.3	72.3	[S6]
B-BCN	0.05 M Na_2SO_4	-0.6	41.9	[S7]
BNQDs@Nb_2CT_x	0.5 M LiClO4	-0.4	66.3	[S8]
BNFC	0.05 M H_2SO_4	-0.4	41	[S9]
**BNCO**	**0.1 M HCl**	**-0.1**	**211.5**	***This work***

Note: BN = boron nitride; BNQDs = boron nitride quantum dots; h-BNNs = hexagonal boron nitride nanosheets.

Table S4 Electrocatalytic NRR performance of different control catalysts in 0.1 M HCl at -0.1 V vs RHE

**Catalysts**	**NH_3 Yield (μg h^{-1} mg_{cat}^{-1})**	**FE (%)**	**Mass-normalized Production rate (mmol h^{-1} g_{cat}^{-1})**
**$BNCO_{(1000)}$**	**211.5**	**34.72**	**12.44**
$BNCO_{(900)}$	164.3	32.26	9.66
$BNCO_{(800)}$	32.38	4.24	1.9
$BNCO_{(1100)}$	161.02	33.43	9.47
BN	86.4	21.36	5.08
NC	29.83	1.24	1.75

Table S5 Double layer capacitance and electrochemical active surface area of all the synthesized catalysts

**Catalyst**	**Double layer capacitance (C_{dl}) in mF cm^{-2}**	**Electrochemical active surface area (ECSA) in cm^2**
$BNCO_{(1000)}$	4.76	8.33
$BNCO_{(1100)}$	4.14	7.24
$BNCO_{(900)}$	3.5	5.47
$BNCO_{(800)}$	0.78	1.33
BN	2.8	4.9
NC	0.41	0.71

Table S6 Atomic % of all the elements comprising the $BNCO_{(1000)}$ catalyst before and after stability tests from XPS analysis

	Boron (B 1s)	Nitrogen (N 1s)	Carbon (C 1s)	Oxygen (O 1s)
**Before stability**	47.71	28.18	15.48	8.63
**After stability**	46.44	27.76	14.23	11.57

Supplementary References

[S1] D.K. Yesudoss, G. Lee, S. Shanmugam, Strong catalyst support interactions in defect-rich γ-Mo_2N nanoparticles loaded 2D-h-BN hybrid for highly selective nitrogen reduction reaction. Appl. Catal. B Environ. **287**, 119952 (2021). https://doi.org/10.1016/j.apcatb.2021.119952

[S2] C. Chen, D. Yan, Y. Wang, Y. Zhou, Y. Zou et al., B-N pairs enriched defective carbon nanosheets for ammonia synthesis with high efficiency. Small **15**(7), 1805029 (2019). https://doi.org/10.1002/smll.201805029

[S3] K. Chu, X. Li, Y. Tian, Q. Li, and Y. Guo, Boron nitride quantum dots/$Ti_3C_2T_x$-MXene heterostructure for efficient electrocatalytic nitrogen fixation. Energy Environ. Mater., (2021). https://doi.org/10.1002/eem2.12247

[S4] Z. Liu, M. Zhang, H. Wang, D. Cang, X. Ji et al., Defective carbon-doped boron nitride nanosheets for highly efficient electrocatalytic conversion of N_2 to NH_3. ACS Sustainable Chem. Eng. **8**(13), 5278-5286 (2020). https://doi.org/10.1021/acssuschemeng.0c00330

[S5] Y. Zhang, H. Du, Y. Ma, L. Ji, H. Guo et al., Hexagonal boron nitride nanosheet for effective ambient N_2 fixation to NH_3. Nano Res. **12**, 919-924 (2019). https://doi.org/10.1007/s12274-019-2323-x

[S6] Q. Li, P. Shen, Y. Tian, X. Li, K. Chu, Metal-free BN quantum dots/graphitic C_3N_4 heterostructure for nitrogen reduction reaction. J. Colloid Interf. Sci. **606**, 204-212 (2022). https://doi.org/10.1016/j.jcis.2021.08.032

[S7] B. Chang, L. Li, D. Shi, H. Jiang, Z. Ai et al., Metal-free boron carbonitride with tunable boron Lewis acid sites for enhanced nitrogen electroreduction to ammonia. Appl. Catal. B Environ. **283**, 119622 (2021). https://doi.org/10.1016/j.apcatb.2020.119622

[S8] K. Chu, X. Li, Q. Li, Y. Guo, H. Zhang et al., Synergistic enhancement of electrocatalytic nitrogen reduction over boron nitride quantum dots decorated Nb_2CT_x-MXene. Small **17**(40), 2102363 (2021). https://doi.org/10.1002/smll.202102363

[S9] Q. Zhang, F. Luo, Y. Ling, S. Xiao, M. Li et al., Identification of functionality of heteroatoms in boron, nitrogen and fluorine ternary-doped carbon as a robust electrocatalyst for nitrogen reduction reaction powered by rechargeable zinc–air batteries. J. Mater. Chem. A **8**(17), 8430-8439 (2020). https://doi.org/10.1039/D0TA01572E
